# Sulfonamide-Derived Dithiocarbamate Gold(I) Complexes Induce the Apoptosis of Colon Cancer Cells by the Activation of Caspase 3 and Redox Imbalance

**DOI:** 10.3390/biomedicines10061437

**Published:** 2022-06-17

**Authors:** Javier Quero, José Carlos Royo, Beatrice Fodor, María Concepción Gimeno, Jesús Osada, María Jesús Rodríguez-Yoldi, Elena Cerrada

**Affiliations:** 1 Departamento de Farmacología y Fisiología y Medicina Legal y Forense, Unidad de Fisiología, Universidad de Zaragoza, 50013 Zaragoza, Spain; 558203@unizar.es (J.Q.); 720204@unizar.es (J.C.R.); 2 Departamento de Química Inorgánica, Instituto de Síntesis Química y Catálisis Homogénea-ISQCH, Universidad de Zaragoza, 50009 Zaragoza, Spain; 822220@unizar.es (B.F.); gimeno@unizar.es (M.C.G.); 3 Departamento de Bioquímica y Biología Molecular y Celular, Universidad de Zaragoza, 50013 Zaragoza, Spain; josada@unizar.es; 4 CIBERobn, ISCIII, 28029 Madrtid, Spain

**Keywords:** dithiocarbamate, sulfonamide, thioredoxin reductase, gold(I), colon cancer, carbonic anhydrase

## Abstract

Two new families of dithiocarbamate gold(I) complexes derived from benzenesulfonamide with phosphine or carbene as ancillary ligands have been synthesized and characterized. In the screening of their in vitro activity on human colon carcinoma cells (Caco-2), we found that the more lipophilic complexes—those with the phosphine PPh_3_—exhibited the highest anticancer activity whilst also displaying significant cancer cell selectivity. [Au(S_2_CNHSO_2_C_6_H_5_)(PPh_3_)] (**1**) and [Au(S_2_CNHSO_2_-p-Me-C_6_H_4_)(IMePropargyl)] (**8**) produce cell death, probably by intrinsic apoptosis (mitochondrial membrane potential modification) and caspase 3 activation, causing cell cycle arrest in the G1 phase with p53 activation. Besides this, both complexes might act as multi-target anticancer drugs, as they inhibit the activity of the enzymes thioredoxin reductase (TrxR) and carbonic anhydrase (CA IX) with the alteration of the redox balance, and show a pro-oxidant effect.

## 1. Introduction

Carbonic anhydrases (CAs) consist of a family of metalloenzymes that catalyse the reversible hydration of carbon dioxide to HCO_3_^−^ and H^+^. CAs are grouped into seven distinct classes (α, β, γ, δ, η, θ, ζ), with the α-class being the best characterized, and being found primarily in vertebrates. Sixteen α-CA isoforms have been described in mammals. The expression of some of these isoforms, namely the transmembrane isoforms CAIX and CAXII, is induced by hypoxia, and they are suggested to promote tumor cell invasion, cell proliferation, and metastasis in colorectal cancer [1]. Both isoforms are overexpressed in an extensive variety of solid tumors which are associated with the hypoxic phenotype, such as colon cancer, among others [2,3], although CAXII is also present under normal conditions in a wide variety of tissues. Therefore, the more specific expression in tumor tissues of CAIX compared to CAXII makes it an interesting target for designing chemotherapeutic agents [4,5,6,7]. Thus, its inhibition could lead to a reduced growth of the primary tumors and metastases, decreasing the population of cancer stem cells [8] and reducing the side effects [6]. To date, several types of molecules that act as carbonic anhydrase inhibitors have been described [9,10,11,12,13]. Among them, sulfonamides (R-SO_2_NH_2_) are best known as inhibitors of human carbonic anhydrases [14]. Besides this, sulfonamides constitute a significant class of chemicals with a wide array of biological activities, such as antibacterial [15,16,17], antifungal [18], antioxidant [19], anti-inflammatory [20] and anticancer activities [21,22,23].

Dithiocarbamates as organosulfur ligands play an important role not only as intermediates in chemical synthesis but also as bioactive compounds with remarkable applications in medicine [24,25]. These include anticancer [26,27,28,29,30], antibacterial [31,32], antitubercular [33], antifungal [34], anti-inflammatory [35] and anti-Alzheimer activities [36,37], among others. Dithiocarbamates have been extensively used as ligands with transition metals because of their antitumor activity. Dithiocarbamate gold derivatives constitute one of the most studied families [38,39,40], particularly gold(III) derivatives, owing to their structural analogy with platinum(II) and their stability in biological environments. Thus, dithiocarbamate gold(III) complexes were found to overcome intrinsic and acquired cisplatin resistance by the inhibition of RNA and DNA synthesis [41,42]. Other putative actions include the inhibition of thioredoxin reductase activity and an increase of ERK1/2 phosphorylation [41]. Glutathione [43]; peptide transporters such as PEPT1 and PEPT2, upregulated in several human tumor cells; [44] and proteasome inhibitors [45,46,47,48] are equally observed targets of dithiocarbamate gold derivatives. Despite these promising antitumor activities, the detailed molecular mechanism remains unknown.

Dithiocarbamates, in addition to sulfonamide derivatives, are also considered to be CA inhibitors [49,50,51,52,53]. Indeed, the combination of both moieties in the same molecule has recently led to sulfonamide–dithiocarbamate hybrids as a potent class of carbonic anhydrase inhibitors [54,55]. In line with this approach, we have addressed the synthesis of dithiocarbamate ligands bearing the benzenesulfonamide moiety, their coordination to gold(I) centers, and their evaluation as potential antitumor agents against the colon cancer cell line Caco-2. We have also studied putative anticancer mechanisms, which include its antiproliferative effect, cell death effectors, and known targets of metallic compounds such as carbonic anhydrase (CA) or thioredoxin reductase (TrxR1).

## 2. Materials and Methods

### 2.1. General

Commercial solvents and reagents were used without further purification. The starting material [AuCl(PPh_3_)], [AuCl(TPPTS)] [56] and [AuBr(IRPropargyl)] (R = Me, Bn) [57] were prepared as previously reported. A Bruker Avance 400 or Bruker ARX 300 spectrometers (Brucker, Billerica, MA, USA) were used to monitor ^1^H, ^13^C{^1^H} and ^31^P{^1^H} chemical shifts (δ, ppm), and were expressed relatively to the solvent peaks in the ^1^H, ^13^C spectra or external 85% H_3_PO_4_ in the ^31^P spectra. The IR spectra were recorded in the range 4000–200 cm^−1^ on a Perkin-Elmer Spectrum 100 spectrophotometer (PerkinElmer Life, Shelton, CT, USA) on solid samples using an attenuated total reflectance accessory. 

### 2.2. Synthesis of the Dithiocarbamates p-RC_6_H_4_SO_2_NHCS_2_Na (R = H, ***L1***; Me, ***L2***; Cl, ***L3***)

To an ice-cold water solution (20 mL) of the corresponding sulfonamide (10 mmol) was added CS_2_ (10 mmol) and a water (10 mL) solution of NaOH (10 mmol). After 6 h of stirring, the solution was filtered and evaporated to dryness. The residue was dissolved in ethanol and filtered through celite, and the solution was reduced to its minimum volume in a vacuum. A white solid was obtained and washed with n-hexane. See Figures S1–S6 for the RMN spectra.

*C_6_H_5_SO_2_NHCS_2_Na* (**L1**): a white solid in a 78% yield. ^1^H NMR (400 MHz, D_2_O, 25 °C) δ(ppm) = 7.73 (dd, 2H, *J_H_*_-*H*_ = 7.6; 1.2 Hz); 7.46–7.41 (m, 3H) ppm. ^13^C{^1^H} NMR (101 MHz): δ(ppm): 146.1 (s, ipso-Ph), 131.0 (s, p-Ph), 128.9 (s, m-Ph), 124.9 (s, o-Ph). IR (cm^−1^): ν(NH) 3303, ν(N-CSS) 1546, ν(SO_2_)_a_ 1206 cm, ν(SO_2_)_s_ 1151, ν(SCS) 978, 1003. The elemental analysis calculated (%) for C_7_H_6_NaNO_2_S_3_ (Pm = 225.298) %C: 32.93 %H: 2.37 %N: 5.49 found: %C: 33.48; %H: 2.58; %N: 6.08. 

*p-Me-C_6_H_4_SO_2_NHCS_2_Na* (**L2**): a white solid in a 70% yield. ^1^H NMR (400 MHz, D_2_O, 25 °C) δ(ppm) = 7.70 and 7.33 (AA′XX′ system, 4H); 2.37 (s, 3H) ppm. ^13^C{^1^H} NMR (101 MHz): δ(ppm) = 143.1 (s, ipso-Ph), 141.9 (s, ipso-Ph), 129.3, 125.0 (s, Ph), 20.45 (s, Me). IR (cm^−1^): ν(NH) 3208, ν(N-CSS) 1598, ν(SO_2_)_a_ 1206, ν(SO_2_)_s_ 1149, ν(SCS) 982, 966. The elemental analysis calculated (%) for C_8_H_8_NaNO_2_S_3_ (Pm = 269.324) %C: 35.67 %H: 2.99 %N: 5.20 found: %C: 36.12; %H: 3.12; %N: 5.75.

*p-Cl-C_6_H_4_SO_2_NHCS_2_Na* (**L3**): a white solid in a 58% yield. ^1^H NMR (400 MHz, D_2_O, 25 °C) δ(ppm) = 7.78 and 7.52 (AA′XX′ system, 4H) ppm. ^13^C{^1^H} NMR (100 MHz): δ(ppm) = 160.9 (CS), 143.8, 137.3 (s, ipso-Ph), 129.5, 127.7 (s, Ph), IR: ν(NH) 3208, ν(N-CSS) 1570, ν(SO_2_)_a_ 1197, ν(SO_2_)_s_ 1147, ν(SCS) 979, 966. The elemental analysis calculated (%) for C_7_H_5_ClNaNO_2_S_3_ (Pm = 288.90) %C: 29.02 %H: 1.74 %N: 4.84 found: %C: 29.56; %H: 2.09; %N: 5.37.

### 2.3. Synthesis of the Complexes [Au(S_2_CNHSO_2_-p-RC_6_H_4_)(PPh_3_)] (R = H, ***1***; Me, ***2***; Cl, ***3***) 

To a dichloromethane solution (10 mL) of [AuClPPh_3_] (0.2 mmol) was added the corresponding dithiocarbamate (0.2 mmol). After 2 h of stirring, the solution was filtered through celite, and the solution was reduced to its minimum volume in a vacuum. A white solid was obtained and washed with n-hexane. See Figures S7–S12 for the RMN spectra.

*[Au(S_2_CNHSO_2_C_6_H_5_)(PPh_3_)]* (**1**): a white solid in a 60% yield. ^1^H NMR (400 MHz, CDCl_3_, 25 °C) δ(ppm) = 8.02 (dd, 2H, *J_H_*_-*H*_ = 7.8; 1.6 Hz); 7.53–7.37 (m, 18H); 3.89 (s, 1H, NH) ppm. ^31^P{^1^H} NMR (162 MHz): δ(ppm) = 32.6 (s). ^13^C{^1^H} NMR (75.4 MHz): δ(ppm) = 147.1 (s, C_ipso_-Ph), 134.1 (d, 6C, C*_o_*, *J* = 13.6 Hz, PPh_3_), 131.9 (d, 3C, C*_p_*, *J* = 2.4 Hz, PPh_3_), 130.5 (s, Cp-Ph), 129.2 (d, 6C, C*_m_*, *J* = 11.8 Hz, PPh_3_), 128.3 (s, C_ipso_, PPh_3_), 128.4 and 126.0 (s, Ph). IR (cm^−1^): ν(NH) 3255, ν(N-CSS) 1582, ν(SO_2_)_a_ 1284, ν(SO_2_)_s_ 1136, ν(SCS) 1010, 997, ν(Au-S) 273. The elemental analysis calculated (%) for C_25_H_21_AuNO_2_PS_3_ (Pm = 691.548) %C: 43.42 %H: 3.06 %N: 2.03 found: %C: 42.96; %H: 3.24; %N: 2.05. 

*[Au(S_2_CNHSO_2_-p-Me-C_6_H_4_)(PPh_3_)]* (**2**): a white solid in a 78% yield. ^1^H NMR (400 MHz, CDCl_3_, 25 °C) δ(ppm) = 7.92 and 7.20 (AA′XX′ system, 4H); 7.55–7.44 (m, 15H, PPh_3_); 3.89 (s, 1H, NH); 2.38 (s, 3H) ppm. ^31^P{^1^H} NMR (162 MHz): δ(ppm) = 32.8 (s). ^13^C{^1^H} NMR (75.4 MHz): δ(ppm) = 145.4 (s, C_ipso_-Ph), 14.8 (s, C_para_-Ph), 134.1 (d, 6C, C*_o_*, *J* = 13.6 Hz, PPh_3_), 131.7 (d, 3C, C*_p_*, *J* = 2.4 Hz, PPh_3_), 129.1 (d, 6C, C*_m_*, *J* = 11.8 Hz, PPh_3_), 128.4 (s, C_ipso_, PPh_3_), 129.0 and 126.0 (s, Ph), 21.4 (s, Me). IR (cm^−1^):ν(NH) 3268, ν(N-CSS) 1598, ν(SO_2_)_a_ 1297, ν(SO_2_)_s_ 1141, ν(SCS) 1020, 997, ν(Au-S) 270 cm^−1^. The elemental analysis calculated (%) for C_26_H_23_AuNO_2_PS_3_ (Pm = 705.574) %C: 44.26 %H:3.29 %N: 1.99 found: %C: 44.16; %H: 3.41; %N: 2.13.

*[Au(S_2_CNHSO_2_-p-Cl-C_6_H_4_)(PPh_3_)]* (**3**): a white solid in a 75% yield. ^1^H NMR (400 MHz, CDCl_3_, 25 °C) δ(ppm) = 7.97 and 7.63 (AA′XX′ system, 4H); 7.58–7.44 (m, 15H, PPh_3_); 3.92 (s, 1H, NH) ppm. ^31^P{^1^H} NMR (162 MHz): δ(ppm) = 32.6 (s). ^13^C{^1^H} NMR (75.4 MHz): δ(ppm) = 145.7 (s, C_ipso_-Ph), 136.5 (s, C-Cl), 134.1 (d, 6C, C*_o_*, *J* = 13.6 Hz, PPh_3_), 132.0 (d, 3C, C*_p_*, *J* = 2.6 Hz, PPh_3_), 129.3 (d, 6C, C*_m_*, *J* = 11.8 Hz, PPh_3_), 128.6 (s, C_ipso_, *J* = 62.4 Hz, PPh_3_), 128.2 and 127.6 (s, Ph). IR (cm^−1^): ν(NH) 3267, ν(N-CSS) 1571, ν(SO_2_)_a_ 1297, ν(SO_2_)_s_ 1144, ν(SCS) 1013, 997, ν(Au-S) 269. The elemental analysis calculated (%) for C_25_H_20_AuClNO_2_PS_3_ (Pm = 725.990) %C: 41.36 %H: 2.78 %N: 1.93 found: %C: 40.86 %H: 2.65 %N: 2.05.

### 2.4. Synthesis of the Complexes [Au(S_2_CNHSO_2_-p-RC_6_H_4_)(TPPTS)] (R = H, ***4***; Me, ***5***; Cl, ***6***) 

The corresponding dithiocarbamate (0.2 mmol) was added to 10 mL of a methanolic solution containing 0.2 mmol [AuClTPPTS]. After 2 h of stirring, the solution was evaporated to dryness and dissolved in dichloromethane. This solution was filtered through celite and reduced to its minimum volume in a vacuum. A white solid was obtained and washed with n-hexane. See Figures S13–S18 for the RMN spectra.

*[Au(S_2_CNHSO_2_C_6_H_5_)(TPPTS)]* (**4**): a white solid in a 74% yield. ^1^H NMR (400 MHz, dmso-d_6_, 25 °C) δ(ppm) = 7.97 and 7.63 (AA′XX′ system, 4H); 7.58–7.44 (m, 15H, PPh_3_); 3.92 (s, 1H, NH) ppm. ^31^P{^1^H} NMR (162 MHz): δ(ppm) = 33.4 (s). ^13^C{^1^H} NMR (101 MHz): δ(ppm) = 7.90–7.80 (m, 8H, Ph + TPPTS), 7.60–7.56 (m, 3H, TPPTS), 7.54–7.51 (m, 2H, Ph), 7.44–7.41 (m, 1H, Ph), 7.38–7.26 (m, 3H, TPPTS). ^31^P{^1^H} NMR (162 MHz): δ(ppm) = 33.4 (s). ^13^C{^1^H} NMR (75.4 MHz): δ(ppm) = 149.6 (d, *J* = 12.1 Hz, C-SO_3_, TPPTS), 134.0 (d, *J* = 12.5 Hz, TPPTS), 131.1 (d, *J* = 16.5 Hz, TPPTS), 129.9 (m, TPPTS), 128.2 (d, *J* = 60 Hz, TPPTS), 129.7 and 129.5 (s, Ph), 21.4 (s, Me). IR (cm^−1^): ν(N-CSS) 1460, ν(SO_2_)_a_ 1310, ν(OSO) 1189, ν(SO) 1036 and 616, ν(SO_2_)_s_ 1145, ν(SCS) 1012, 995, ν(Au-S) 270 cm^−1^. The elemental analysis calculated (%) for C_25_H_18_AuNNa_3_O_11_S_6_ (Pm = 997.68) %C: 30.10 %H: 1.82 %N: 1.40 found: %C: 29.92 %H: 2.24 %N: 0.9.

*[Au(S_2_CNHSO_2_-p-Me-C_6_H_4_)(TPPTS)]* (**5**): a white solid in a 70% yield. ^1^H NMR (400 MHz, dmso-d_6_, 25 °C) δ(ppm) = 7.87–7.74 (m, 8H, Ph + TPPTS), 7.62–7.58 (m, 3H, TPPTS), 7.40–7.32 (m, 5H, TPPTS + Ph), 2.34 (s, 3H, Me). ^31^P{^1^H} NMR (162 MHz): δ(ppm) = 33.3 (s). ^13^C{^1^H} NMR (101 MHz): δ(ppm) = 149.6 (d, *J* = 12.1 Hz, C-SO_3_, TPPTS), 134.1 (d, *J* = 12.5 Hz, TPPTS), 131.1 (d, *J* = 16.5 Hz, TPPTS), 130.5 (s, C_p_-Ph), 129.8 (m, TPPTS), 127.8 and 125.7 (s, Ph). IR (cm^−1^): ν(N-CSS) 1465, ν(SO_2_)_a_ 1290, ν(OSO) 1189, ν(SO) 1036 and 617, ν(SO_2_)_s_ 1148, ν(SCS) 1013, 996, ν(Au-S) 275 cm^−1^. The elemental analysis calculated (%) for C_26_H_20_AuNNa_3_O_11_S_6_ (Pm = 1011.71) %C: 30.67 %H: 1.99 %N: 1.38 found: %C: 29.92 %H: 2.44 %N: 0.8.

*[Au(S_2_CNHSO_2_-p-Cl-C_6_H_4_)(TPPTS)]* (**6**): a white solid in a 75% yield. ^1^H NMR (400 MHz, dmso-d_6_, 25 °C) δ(ppm) = 7.89 and 7.61 (AA′XX′ system, 4H, Ph); 7.84–7.74 (m, 6H, TPPTS), 7.59–7.51 (m, 3H, TPPTS), 7.36–7.242 (m, 3H, TPPTS). ^31^P{^1^H} NMR (162 MHz): δ(ppm) = 33.5 (s). IR (cm^−1^): ν(N-CSS) 1465, ν(SO_2_)_a_ 1308, ν(OSO) 1189, ν(SO) 1036 and 616 ν(SO_2_)_s_ 1147, ν(SCS) 1011, 993, ν(Au-S) 270 cm^−1^. The elemental analysis calculated (%) for C_25_H_17_AuClNNa_3_O_11_S_6_ (Pm = 1032.13) %C: 29.09 %H: 1.66 %N: 1.36 found: %C: 28.57 %H: 2.04 %N: 0.84.

### 2.5. Synthesis of the Complexes [Au(S_2_CNHSO_2_-p-RC_6_H_4_)(IR″Propargyl)] (R = H, R″ = Me, ***7***, R″ = Bn, ***9***; R = Me, R″ = Me, ***8***; R″ = Bn, ***10***)

To a dichloromethane solution (10 mL) of [AuCl(NHC)] (0.2 mmol) was added the corresponding dithiocarbamate (0.2 mmol). After 1 h of stirring, the solution was filtered through celite, and the solution was reduced to its minimum volume in a vacuum. A white solid was obtained after the addition of pentane. See Figures S19–S26 for the RMN spectra.

*[Au(S_2_CNHSO_2_C_6_H_5_)(IMePropargyl)]* (**7**): a cream solid in a 60% yield. ^1^H NMR (400 MHz, dmso-d_6_, 25 °C) δ(ppm) = 7.85–7.82 (m, 2H, Ph), 7.60–7.56 (m, 3H, Ph), 7.54 (d, *J* = 1.9 Hz, 1H, CH_imidazole_), 7.48 (d, *J* = 1.9 Hz, 1H, CH_imidazole_), 7.34 (s_br_, 1H, -NH-), 5.00 (d, *J* = 2.6 Hz, 2H, *CH_2_*), 3.77 (s, 3H, CH_3_), 3.59 (t, *J* = 2.6 Hz, 1H, C≡CH). ^13^C{^1^H} APT RMN (101 MHz) δ: 173.0 (s, N*C*HN), 144.6(s, ipso-Ph), 132.2 (s, p-Ph), 129.38 (s, m-Ph), 126.0 (s, o-Ph), 123.6 (s, CH_im_), 121.75 (s, CH_im_), 78.6 (s, C≡CH), 77.7 (s, C≡CH), 40.3 (s, *CH_2_*-C≡CH), 38.2 (s, *CH_3_*). IR (cm^−1^): ν(-NH-) 3352, ν(C≡CH) 3257, ν(C≡C) 2123, ν(N-CSS) 1566, ν(SO_2_, a) 1288, ν(SO_2_, s) 1156, ν(CSS) 1010, 997, ν(Au-S): 263. The elemental analysis calculated (%) for C_14_H_14_AuN_3_O_2_S_3_ (Pm = 549.43) %C: 30.61 %H: 2.57 %N: 7.65 found: %C: 29.92 %H: 2.80 %N: 7.24.

*[Au(S_2_CNHSO_2_-p-Me-C_6_H_4_)(IMePropargyl)]* (**8**): a cream solid in an 89% yield. ^1^H NMR (400 MHz, dmso-d_6_, 25 °C) δ(ppm) = 7.71 (d, *J* = 8.0 Hz, 2H, Ph), 7.54 (d, *J* = 1.9 Hz, 1H, CH_im_), 7.48 (d, *J* = 1.9 Hz, 1H, CH_im_), 7.36 (d, *J* = 8.0 Hz, 2H, Ph), 7.25 (s, br, 1H, -NH-), 5.00 (d, *J* = 2.6 Hz, 2H, *CH_2_*), 3.77 (s, 3H, CH_3_), 3.59 (t, *J* = 2.6 Hz, 1H, C≡CH), 2.37 (s, 3H, CH_3_). ^13^C{^1^H} NMR (101 MHz): δ(ppm) = 172.51 (s, N*C*HN), 141.83 (s, p-Ph), 141.38 (s, ipso-Ph), 129.24 (s, m-Ph), 125.53 (s, o-Ph), 123.02 (s, CH_imidazole_), 121.25 (s, CH_imidazole_), 78.12 (s C≡CH), 77.21 (s, C≡CH), 39.78 (s, *CH_2_*-C≡CH), 37.67 (s, CH_3_), 20.74 (s, CH_3_-Ph). IR (cm^−1^): (-NH-) 3358; ν(C≡CH) 3261, ν(N-CSS) 1561, ν(SO_2_, a) 1299, ν(SO_2_, s) 1164, ν(CSS) 1003, 997, ν(Au-S): 255. The elemental analysis calculated (%) for C_15_H_16_AuN_3_O_2_S_3_ (Pm = 563.46) %C: 31.97 %H: 2.86 %N: 7.46 found: %C: 30.40 %H: 2.82 %N: 7.44.

*[Au(S_2_CNHSO_2_C_6_H_5_)(IBnPropargyl)]* (**9**): a cream solid in an 84% yield. ^1^H NMR (400 MHz, dmso-d_6_, 25 °C) δ(ppm) = 7.85–7.81 (m, 2H, Ph), 7.62–7.57 (m, 5H, 3H Ph + 2 CH_im_), 7.39–7.36 (m, 6H, Ph_Bn_+ 1H, -NH-), 5.37 (s, 2H, *CH_2_*-Ph_Bn_), 5.03 (d, *J* = 2.6 Hz, 2H, *CH_2_*), 3.62 (t, *J* = 2.6 Hz, 1H, C≡CH). ^13^C{^1^H} APT RMN (101 MHz) δ: 201.29 (s, S*C*S), 172.57 (s, N*C*HN), 144.11 (s, ipso-Ph), 136.43 (s, ipso-Ph_Bn_), 131.76 (s, p-Ph), 128.90 (s, m-Ph), 128.76 (s, m-Ph_Bn_), 127.64 (s, o-Ph_Bn_), 127.53 (s, p-Ph_Bn_), 125.54 (s, o-Ph), 122.09 (s, CH_imidazole_), 121.86 (s, CH_imidazole_), 77.96 (s C≡CH), 77.36 (s, C≡CH), 53.82 (s, *CH_2_*-Ph_Bn_), 39.37 (s, *CH_2_*-C≡CH). IR (cm^−1^): ν(-NH-) 3346; ν(C≡CH) 3256; ν(C≡C) 2131; ν(N-CSS) 1559; ν(SO_2_, a) 1297; ν(SO_2_, s) 1156; ν(CSS) 1002, 996, ν(Au-S): 270. The elemental analysis calculated (%) for C_20_H_18_AuN_3_O_2_S_3_ (Pm = 625.53) %C: 38.40 %H: 2.90 %N: 6.72 found: %C: 37.89 %H: 2.95 %N: 6.68.

*[Au(S_2_CNHSO_2_-p-Me-C_6_H_4_)(IBnPropargyl)]* (**10**): a cream solid in an 80% yield. ^1^H NMR (400 MHz, CDCl_3_, 25 °C) δ(ppm) = 7.72 (d, *J* = 8.0 Hz, 2H, Ph), 7.62 (d, *J* = 1.9 Hz, 1H, CH_imidazole_), 7.59 (d, *J* = 1.9 Hz, 1H, CH_imidazole_), 7.38–7.35 (m, 7H, 2Ph + Ph_Bn_), 7.27 (s br, 1H, -NH-), 5.37 (s, 2H, *CH_2_*-Ph_Bn_), 5.03 (d, *J* = 2.6 Hz, 2H, *CH_2_*-C≡CH), 3.63 (t, *J* = 2.6 Hz, 1H, C≡CH), 2.37 (s, 3H, CH_3_). ^13^C{^1^H} APT RMN (101 MHz) δ: 172.51 (s, N*C*HN), 141.82 (s, ipso-Ph), 141.36 (s, p-Ph), 136.32 (s, ipso-Ph (Bn)), 129.27 (s, m-Ph), 128.76 (s, m-Ph_Bn_), 128.16 (s, o-Ph_Bn_), 127.64 (s, p-Ph_Bn_), 125.58 (s, o-Ph), 122.09 (s, CH_imidazole_), 121.86 (s, CH_imidazole_), 78.00 (s C≡CH), 77.35 (s, C≡CH), 53.80 (s, *CH_2_*-Ph_Bn_), 40.08 (s, *CH_2_*-C≡CH), 20.89 (s, CH_3_). IR (cm^−1^): ν(-NH-) 3355, ν(C≡CH) 3260, ν(C≡C) 2137, ν(N-CSS) 1527, ν(SO_2_, a) 1298, ν(SO_2_, s) 1156, ν(CSS) 1005, 995, ν(Au-S): 304. The elemental analysis calculated (%) for C_21_H_20_AuN_3_O_2_S_3_ (Pm = 639.56) %C: 39.44 %H: 3.15 %N: 6.57 found: %C: 38.93 %H: 3.17 %N: 6.16.

### 2.6. Distribution Coefficient (Log P_7.4_)

The n-octanol−water coefficients of the complexes were determined using a shake-flask method, as reported previously [58]. Briefly, 100 mL buffered-saline (0.15 M NaCl, 10 mM phosphate [PO_4_^3−^] buffer, pH 7.4) and 100 mL n-octanol were shaken for 72 h. The addition of 1 mg of the complexes followed by the centrifugation and separation of both phases gave two solutions, which were analysed by UV absorbance spectroscopy to determine their concentration in each phase. The corresponding value of log P was defined as log{[compound(organic)]/[compound(aqueous)]}.

### 2.7. Solution Chemistry

The stability of the new derivatives was analyzed by absorption UV spectroscopy. The spectra of the complexes were recorded on a Thermo Scientific spectrophotometer (Thermo Fisher Scientific, Waltham, MA, USA). Solutions of the complexes (5 × 10^−5^ M) in PBS (pH 7.4) were prepared from their stock solutions in dimethylsulfoxide (10 mM). The final concentration of DMSO in the test cell was 0.5%. The samples were then incubated at 37 °C and their UV spectra were registered over 24 h. 

### 2.8. Culture, Treatment and Cytotoxicity Determination in the Cells

The human Caco-2 cell line (TC7 clone) was kindly provided by Dr. Edith Brot-Laroche (Université Pierre et Marie Curie-Paris 6, UMR S 872, Les Cordeliers, France). Human epithelial fibroblast cells (NHDF-Ad, Lonza^®®^, Porriño, Spain) were kindly provided by Dr. Gracia Mendoza (Aragon Health Research Institute, IIS Aragón, Spain). Caco-2 cells (passages 40–70) and fibroblasts (passages 10–30) were maintained in a humidified atmosphere of 5% CO_2_ at 37 °C and grown in medium following the method used by Quero et al. [59]. The Caco-2 cells were sub-cultured in 25 cm^2^ plastic flasks at a density of 1.2 × 10^4^ cells/cm^2^, while the fibroblasts were sub-cultured on 75 cm^2^ plastic flasks at a density of 1 × 10^4^ cells/cm^2^. The culture medium was replaced every 2 days.

The gold complexes were diluted in dimethyl sulfoxide (DMSO) as a 10 mM stock solution, and then diluted in cell culture medium at the desired concentrations. 

For the cytotoxicity screening assays, Caco-2 cells were seeded in 96-well plates at a density of 4 × 10^3^ cells/well. The culture medium was replaced with medium containing the drug panel 24 h post-seeding, and the cells were incubated for 24, 48 and 72 h. An initial range of complex concentrations of 1.25–20 µM in undifferentiated Caco-2 and 5–50 µM in differentiated Caco-2 were used to determine the IC_50_ values. For specific determinations in undifferentiated Caco-2 cells, the range of concentrations was adjusted depending on the assayed compound: 0.5–2.5 μM (complexes 1, 2 and 3), 15–40 μM (complex 4), 35–60 μM (complex 5), 10–50 μM (complex 6), 20–40 μM (complex 7), 1.25–7.5 μM (complex 8), 2.5–12.5 μM (complex 9), and 1.25–20 μM (complex 10). In differentiated Caco-2 and fibroblasts, culture medium containing metal gold complexes was added 15 days post-seeding, and the cells were incubated for 72 h. The antiproliferative effect was measured using an MTT assay, as previously described by Mármol et al. [60]. The absorbance at 540/620 nm was measured using a SPECTROstar Nano (BMG Labtech, Ortenberg, Germany). In order to determine the selectivity index (SI), the IC_50_ value of the differentiated Caco-2 cells was divided by IC_50_ value in undifferentiated Caco-2, obtaining the ratio of the normal/cancerous cells’ toxicity.

### 2.9. Apoptosis Measurement 

Caco-2 cells were seeded in 75 cm^2^ flasks at a density of 1 × 10^4^ cells/cm^2^, then exposed to the drug panel for 48 or 72 h depending on the compound tested, and them stained with annexin V-FITC and sodium iodide propidio according to the manufacturer’s instructions. The cells were then transferred to flow cytometry tubes and washed twice with phosphate-buffered saline (PBS); they were then resuspended in 100 µL annexing V binding buffer (100 mM Hepes/NaOH pH 7.4, 140 nM NaCl, 2.5 mM CaCl_2_). In total, 5 µL annexin V-FITC and 5 µL propidium iodide (PI) were added to the tube. After 15 min of incubation at room temperature protected from light, 400 µL annexin binding buffer was added to each sample, and the signal intensity was analyzed within 1 h with Beckman Coulter Gallios (Brea, CA, USA). The data were analyzed with BD FACSDivaTM [59]. 

### 2.10. Propidium Iodide Staining of the DNA Content and Cell Cycle Analysis

Once the cells were seeded in 25 cm^2^ flasks at a density of 1 × 10^4^ cells/cm^2^, they were exposed, after 24 post-sendings, to the drug panel for 48 or 72 h. The cells were fixed according to the previously described methods [59,60] before being stained with 50 μg/mL PI solution. The signal intensity was analysed with a Beckman Coulter Gallios (Brea, CA, USA) equipped with a blue solid diode laser (488 nm) and a red solid diode laser (635 nm), collecting fluorescence with a 620 nm-long pass filter. The cell distribution was displayed on a linear scale, and was analysed with ModFit LT^TM^ verity software, which determined the percentage of cells in every cycle phase.

### 2.11. Mitochondrial Membrane Potential Assay 

Caco-2 cells were seeded in 75 cm^2^ flasks at a density of 1 × 10^4^ cells/cm^2^, and the 24-h post-seeding medium was replaced with medium containing the drug panel; it was then incubated for 48 or 72 h. The cells were then washed twice and resuspended in PBS at a concentration of 10^6^ cells/mL before incubation with 5 µL of 10 µM 1,1′,3,3,3′-hexamethylindodicarbo-cyanine iodide (DilC1). After 15 min of incubation at 37 °C, 400 µL PBS was added, and the fluorescence was measured by flow cytometry at an excitation wavelength of 633 nm and an emission of 658 nm with a Beckman Coulter Gallios (Brea, CA, USA) equipped with a blue solid diode laser (488 nm) and a red solid diode laser (635 nm) [59].

### 2.12. Determination of the Caspase 3 and p53 Proteins

Caco-2 cells were seeded in a 75 cm^2^ flask at a density of 1 × 10^4^ cells/cm^2^; 24 h later, they were exposed to the drug panel for 48 or 72 h. Then, the cells were collected and processed as previously described [59,61]. Finally, 50 µL of every sample was incubated with 5 µL anti-active caspase-3 (BD Pharmigen, San Diego, CA, USA, Clone C92-605) and 5 µL p53 antibody (Miltenyi, Auburn, CA, USA, Clone REA609). The fluorescence was measured by flow cytometry using a Beckman Coulter Gallios (Brea, CA, USA) equipped with a blue solid diode laser (488 nm) and a red solid diode laser (635 nm). For the caspase-3 determination, the excitation wavelength was set at 488 nm and the emission was set at 525 nm; for the p53 analysis, the excitation was set at 635 nm and the emission was set at 660 nm.

### 2.13. Thioredoxin Reductase 1 (TrxR1) Activity Assay

The in vitro interaction of metal complexes with the enzyme thioredoxin reductase was analyzed using a thioredoxin reductase assay kit (Sigma, CS0170, St Louis, MO, USA). The procedure instructions in the kit were followed, though the rat liver thioredoxin reductase was replaced with recombinant human enzyme TrxR1 (SRP 6081, Sigma, St Louis, MO, USA). The reaction was initiated by adding DTNB (100 mM), and conversion to TNB^−^ was monitored at 412 nm every 30 s for 22 min using a SPECTROstar Nano (BMG Labtech, Ortenberg, Germany) multiplate reader. The results were expressed as a percentage of the TrxR activity with respect to the control.

### 2.14. Carbonic Anhydrase (CA) Activity

The in vitro effect of metal complexes on the enzyme carbonic anhydrase IX was analyzed by monitoring conversion of 4-nitrophenyl acetate (4-NPA) to 4-nitrophenol. The tests were carried out in 96-well plates. Gold complexes (4 µL) were incubated with 4 µL recombinant human carbonic anhydrase IX (Sigma SRP6483, St Louis, MO, USA) (50 µg/mL) at 25 °C for 5 min. PBS at pH 7.35 (Sigma D1408, St Louis, MO, USA) was added to reach a final volume of 90 µL. The reaction was started by adding 10 µL 4-NPA (0.5 mM in methanol), and the absorbance at 405 nm was monitored every 30 s for 1 h using a SPECTROstar Nano (BMG Labtech, Ortenberg, Germany) multiplate reader. The results were expressed as a percentage of CA-IX activity with respect to the control. The potency of the inhibtion referred to the incubation in the presence of indisulam (E7070, *N*-(3-chloro-7-indolyl)-1,4-benzenedisulfonamide) (Sigma SML1225, St Louis, MO, USA), which is a specific inhibitor of carbonic anhydrase isoforms IX and XII.

### 2.15. Intracellular Levels of Reactive Oxygen Species (ROS)

After the Caco-2 cells were seeded in a 96-well plate at a density of 4 × 10^3^ cells/well, the intracellular ROS levels were determined with a dichlorofluorescein assay [62], following the protocol used by Quero et al. [59]. The cells were exposed to the drug panel for 1, 3 and 24 h, and then incubated with 20 µM 2′-7′-dichlorofluorescein diacetate (DCFH-DA) (Merck KGaA, Darmstadt, Germany) in DMEM. The generation of oxidized derivative DCF was monitored by measuring the increase of the fluorescence for 1 h, at an emission wavelength of 520 nm and an excitation of 485 nm, using a FLUOstar Omega (BMG Labtech, Ortenberg, Germany) multiplate reader. The results were expressed as percentage of the fluorescence with respect to the control, considering the fluorescence intensity as a reflection of the intracellular ROS levels. 

### 2.16. Statistical Analysis

The statistical analysis and the creation of the graphics were performed using GraphPad Prism Version 5.02 program on a PC computer (La Jolla, CA USA). All of the assays were performed at least three times. The data were presented as the mean ± SD using one-way analysis of variance (ANOVA). The significant differences at *p* < 0.05 were compared using Bonferroni’s Multiple Comparison Test. 

## 3. Results and Discussion

### 3.1. Chemical Studies

**Scheme 1:**
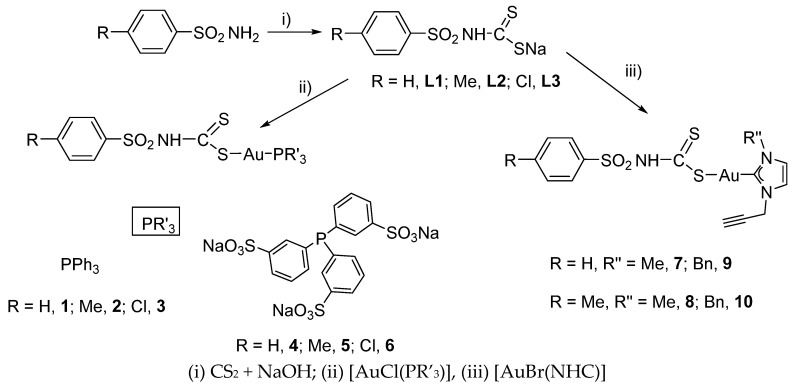
Synthesis of dithiocarbamates and the corresponding gold derivatives.

The dithiocarbamate sodium salts **L1–3** (Scheme 1) bearing the sulphonamide moiety were readily obtained by the reaction of the primary amines benzenesulfonamide, 4-methylbenzenesulfonamide and 4-chlorobenzenesulfonamide with CS_2_ in the presence of sodium hydroxide as a base. The salts were characterized by NMR and IR spectroscopy. Their ^1^H NMR spectra display the corresponding multiplets due to the phenyl ring in the case of **L1** and the AA′XX′ system in the cases of *para*-benzene-substituted **L2** and **L3**. Their IR spectra show the most significant bands, such as the bands assigned to ν(NH), ν_sym_(SO_2_), and ν_as_(SO_2_), with the later displaced to lower wavenumbers in comparison with the sulfonamide starting material, ν(N-CSS) and ν(C-S) vibrations.

The addition of [AuCl(PR_3_)] (PR_3_ = PPh_3_ or TPPTS, trisulfonated triphenylphosphine sodium salt) or [AuBr(NHC)] (NHC = IRPropargyl; R = Me, Bn) to the dithiocarbamate-sulfonamide sodium salts **L1–3**, afforded the isolation of the corresponding phosphane dithiocarbamate gold(I) complexes **1**–**3** (with PPh_3_) and **4**–**6** (with TPPTS) and the carbene dithiocarbamate homologues **7**–**10** as air- and moisture-stable solids at room temperature.

Their ^1^H NMR spectra display similar signals to those of the starting ligands shifted to a low field due to the gold coordination. In the case of the NHC derivatives (**7**–**10**), the formation of the dithiocarbamate carbene species was confirmed by the disappearance of the C2-H (N*C*HN) imidazoleium proton in the ^1^H NMR spectra (at around 9.3 ppm), the downfield shift of the carbene carbon atom nuclei in their ^13^C{^1^H} NMR spectra to around 170 ppm, and a signal ca. 200 ppm assigned to the N*C*S_2_ carbon atom. 

The presence of dithiocarbamate ligands was also confirmed by the appearance of the typical bands in three regions of the IR [63]. Thus, there is a band in the 1450–1580 cm^−1^ region, which is primarily associated with the thioureide band due to the ν(N-CSS) vibration; besides this, there was a band in the 940–1060 cm^−1^ region, associated with ν(C-S) vibrations, and a band in the 250–420 cm^−1^ region associated with ν(Au-S) vibrations. The presence of a unique band in the region 940–1060 cm^−1^ due to the –CSS moiety is commonly assumed to a symmetrical bonding of the dithiocarbamate ligand, which acts as a chelate ligand. A split band is observed in the new derivatives, which indicates an asymmetrically bidentate ligand or a monodentate ligand [64,65]. Examples of dithiocarbamate gold(I) derivatives have previously been described in the literature, in which an asymmetrically bidentate mode of coordination of the ligand—due to short Au…S interactions—was confirmed by the X-ray structure [66].

The stability of the new complexes was studied over 24 h at 37 °C using UV-vis absorption spectroscopy in a PBS solution (pH = 7.4). Solutions suitable for spectrophotometric analysis were prepared by the dilution of dimethylsulfoxide (DMSO) stock solutions of the complexes in PBS buffer. The spectra of the corresponding complexes (Figure S27) show an intense absorption band at ca. 210 nm and one lower-energy absorption band with low intensity at around 260 nm, which could be assigned to π→π* intraligand transitions. These bands remain without any changes in shape or displacement in the absorbance maximum (with no apparent red or blue shift) in all of the new derivatives, in addition to lacking absorbance at around 500 nm—due to the gold nanoparticle formation—over 24 h, implying the substantial stability of the chromophore under physiological conditions.

### 3.2. Biological Studies 

#### 3.2.1. Antiproliferative Activity of Gold Complexes 

The antiproliferative properties of these dithiocarbamate gold(I) complexes derived from sulfonamides were analyzed using the human carcinoma cell line Caco-2. The cytotoxic effect of the gold complexes on these cells was evaluated by the determination of the IC_50_ values (a necessary concentration to reduce the two-fold cell viability) (Table 1). 

Among all of the tested complexes, the combination of the benzenesulfonamide moeity with the triphenylphosphine ligand (Table 2) seems to be the most cytotoxic, as it displayed the lowest IC_50_ values, with [Au(S_2_CNHSO_2_C_6_H_5_)(PPh_3_)] (**1**) being the most toxic. Besides this, these complexes displayed the highest lipophilic character, with log P_7.4_ values close to 1 (Table 1). No significant differences in the IC_50_ values were observed between these three complexes, suggesting that the substitution on the benzene ring in the dithiocarbamate unit seems to be irrelevant. 

On the other hand, the complexes with the water-soluble triphenylphosphine-trisulfonated (TPPTS) ligand, which in fact are the most hydrophilic derivatives (with log *p* values < 0), display much higher IC_50_ values, ranging from 30 to 50 μM. (Table 2), with complex **4** being the significantly least cytotoxic, with an IC_50_ of around 50 μM and no significant differences between complexes **5** and **6**.

In the case of carbene benzenesulfonamide complexes, the presence of the benzyl substituent in the NHC ring (complex **9**) instead of methyl (complex **7**) improves their cytotoxicity (Table 1). However, the presence of the methyl group in both ligands, benzenesulfonamide and carbene (complex **8**), results in a more effective combination than the use of methyl and benzyl, respectively (complex **10**). Curiosuly, [Au(S_2_CNHSO_2_C_6_H_5_)(IMePropargyl)] (**7**) is the least-active derivative in the carbene dithiocarbamate family, despite the similarity in its structure (Table 1). In general, there is no linear correlation between IC_50_ values and log P_7.5_; nevertheless, the most hydrophilic complexes, those with TPPTS, are the least cytotoxic.

**Figure 1:**
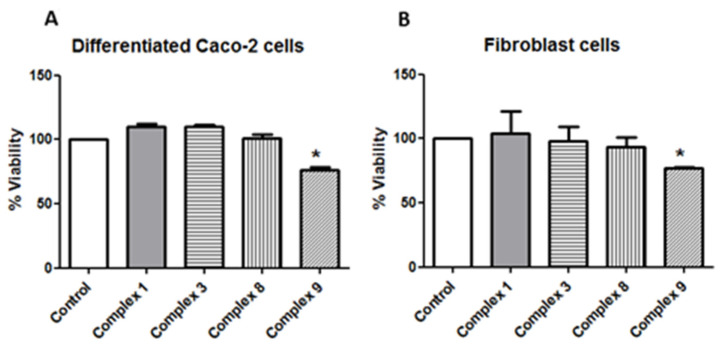
(**A**) Viability percentages obtained after the treatment of differentiated Caco-2 cells with complexes **1** (1.14 μM), **3** (1.7 μM), **8** (7.22 μM), and **9** (8.56 μM) for 72 h. (**B**) Viability percentages obtained after treating epithelial fibroblasts with complexes **1** (1.14 μM), **3** (1.7 μM), **8** (7.22 μM), and **9** (8.56 μM). * *p* < 0.05 vs the respective control.

One of the problems with chemotherapeutic agents is the lack of selectivity for cancer cells compared to healthy ones, which can lead to side effects. For this reason, we evaluated their toxicity in two models of healthy cells at their IC_50_ values: human epithelial fibroblasts and differentiated Caco-2 cells with complexes **1**, **3**, **8** and **9** (Figure 1).

The results showed that only complex **9** was able to significantly decrease the cell viability (approximately 39%) both in fibroblasts and in differentiated Caco-2 cells, which involves a *per se* effect. On the other hand, complexes **1**, **3** and **8** did not compromise the viability of healthy cells at their IC_50_ concentration. Besides this, the IC_50_ values of these compounds calculated in the differentiated Caco-2 cells were significantly higher compared to the undifferentiated cells (cancerous ones) (Table 2), which points to a specificity towards cancer cells (Table 1).

**Figure 2:**
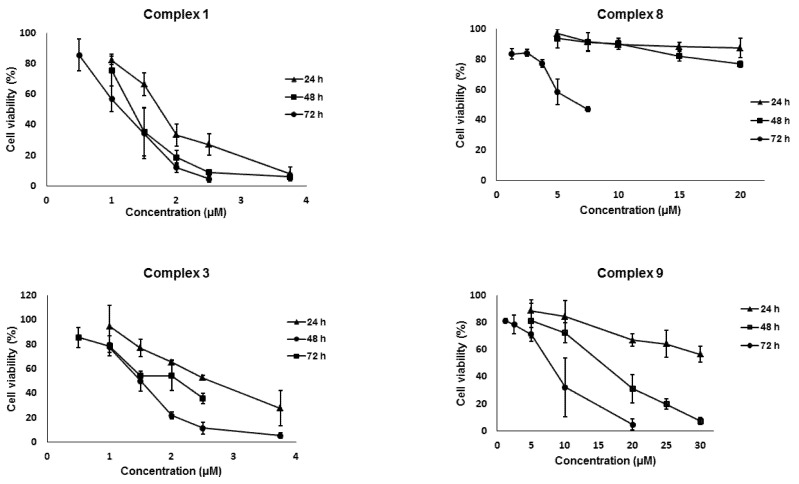
Dose–response curves of the Caco-2 cell viability percentage of metal complexes at different concentrations and incubation times.

We also evaluated the toxicity of the compounds after shorter times (24 and 48 h). The results showed a time- and concentration-dependent curve (Figure 2). The corresponding IC_50_ values are depicted in Table 3. Thus, there were significant differences in the IC_50_ values obtained for complexes **1** and **3** after 24 h of incubation with respect to 48 and 72 h, which indicates that after 48 h of incubation the effect is time-independent. However, the cytotoxicity of complexes **8** and **9** increased dramatically after 72 h, involving a time-dependent effect. In fact, both complexes (**8** and **9**) displayed a band in the UV-vis spectra at ca. 250 nm (Figure S27) that disappeared over time, which could explain such an increase in its cytotoxicity, due to a period of activation.

In order to gain some insights into their mechanism of action in cancer cells, we chose the complexes [Au(S_2_CNHSO_2_C_6_H_5_)(PPh_3_)] **(1)** from the PPh_3_ family and [Au(S_2_CNHSO_2_-p-Me-C_6_H_4_)(IMePropargyl)] **(8)** from the carbene famliy, which are the most active derivatives according to their selectivity indexes.

#### 3.2.2. Type of Cell Death Produced by the Metal Complexes

**Figure 3:**
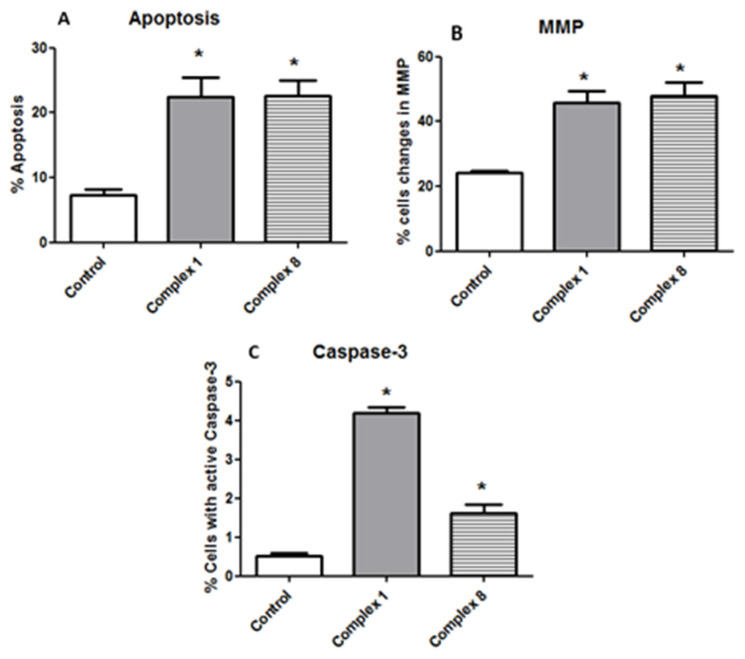
Caco-2 cell death studies by flow cytometry after incubation with complexes **1** (1.14 µM) and **8** (7.22 µM) for 48 and 72 h, respectively. (**A**) The results of the apoptotic state are expressed as a percentage. (**B**) The percentage of Caco-2 cells with disturbed mitochondrial membrane potential (MMP). (**C**) The percentage of Caco-2 cells with the presence of active caspase-3. * *p* < 0.05 compared to the respective control.

Because these new gold complexes produced a decrease in cell proliferation, we performed assays to determine whether complexes **1** and **8** produced cell death by necrosis or apoptosis. Double staining with annexin V/PI showed that both gold complexes were capable of inducing apoptosis in Caco-2 cells after 48 h (complex **1**) or 72 h (complex **8**) of incubation, with the former being more effective even at the shorter incubation time (Figure 3A).

The depolarization of the mitochondrial membrane (MMP) occurs in apoptosis, which induces a series of structural changes that ultimately lead to the release of cytochrome c. The DilC1 [67] staining results showed that both complex **1** and **8** induced similar mitochondrial depolarization (Figure 3B). Mitochondrial depolarization and the release of cytochrome c finally lead to the activation of caspase-3, which is the executor of the apoptotic process. Flow cytometry determinations confirmed the previous results, as incubation with both derivatives increased the percentage of cells with active caspase-3 (Figure 3C). However, complex **1** proved to be more effective, suggesting that the presence of the triphenylphosphine could probably affect other apoptotic pathways besides the mitochondrial one. The presence of the AuPPh_3_ moiety in **1** probably favors its cellular uptake enhancing its effect, as has been observed in other complexes with the same unit [68,69].

#### 3.2.3. Effect of the Gold Complex on the Cell Cycle

**Figure 4:**
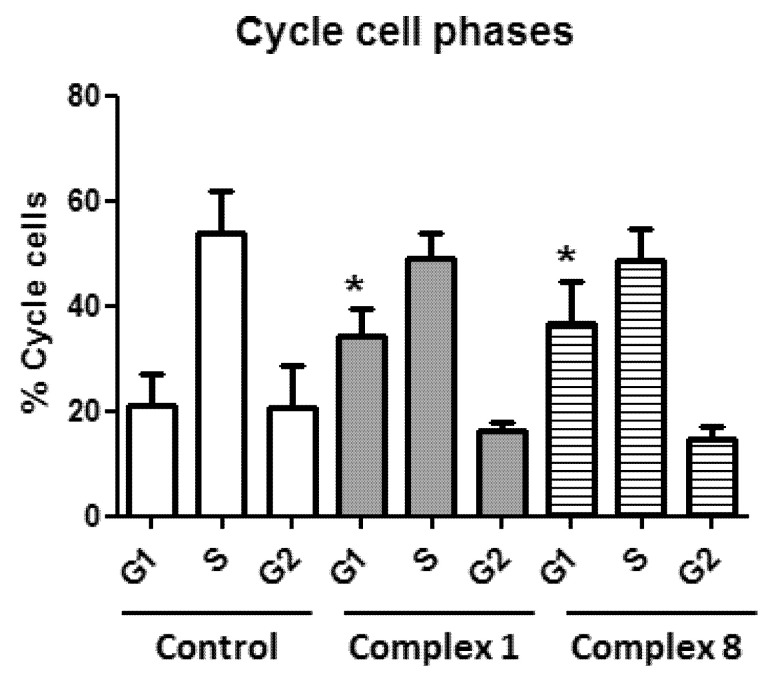
Percentages of the Caco-2 cycle cell phase studies by flow cytometry after incubation with complexes **1** (1.14 µM) and **8** (7.22 µM) for 48 and 72 h, respectively, with the distribution of the cells along the cell cycle phases. * *p* < 0.05 compared to the control.

Apoptosis processes are usually accompanied by alterations in the cell cycle, and previous studies have suggested that gold-complex-treated cells may arrest the cell cycle in some of its phases [59,70,71,72]. Flow cytometry analysis showed that after 48h (complex **1**) or 72 h (complex **8**) of incubation time, both derivatives significantly increased the number of cells arrested in the G1 phase, with a slight decrease in the S phase (Figure 4).

**Figure 5:**
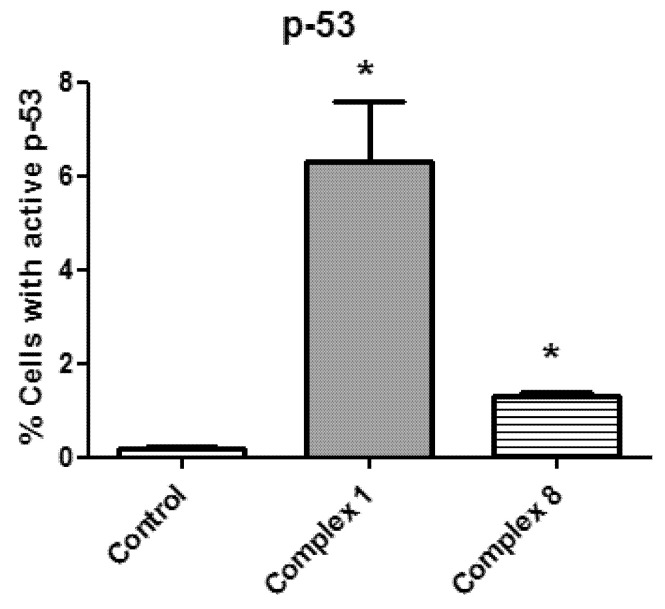
Percentage of Caco-2 cells with the presence of active p53. Studies by flow cytometry after incubation with complexes **1** (1.14 µM) and **8** (7.22 µM) for 48 and 72 h, respectively. * *p* < 0.05 compared to the respective control.

An important factor in cell cycle control is the p53 protein [73]. Thus, the treatment of cancer cells with drugs can induce stress in them, which causes the activation of the p53 protein, which is one of the most important tumor suppressors [74,75]. Furthermore, p53 can induce apoptotic cell death in response to DNA damage [76]. Taking all of this into consideration, the presence of p53 in the cells treated with complexes **1** and **8** was assayed, and the results showed a significant increase in cells with active p53 compared to the untreated cells. (Figure 5). Likewise, a greater effect of [Au(S_2_CNHSO_2_C_6_H_5_)(PPh_3_)] (**1**) was observed in accordance with the data obtained for caspase 3 (Figure 3C).

#### 3.2.4. Thioredoxin Reductase and Carbonic Anhydrase IX as Potential Targets

**Figure 6:**
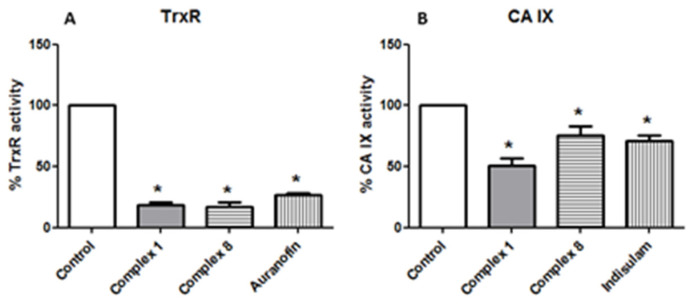
(**A**) Determination of the reductase activity of recombinant TrxR1 after in vitro incubation with complex **1** (1.14 μM) and complex **8** (7.22 μM) for 5 min. (**B**) Determination of the esterase activity of the enzyme carbonic anhydrase IX after 5 min of incubation with complexes **1** and **8** (30 μM) and indisulam (60 μM). * *p* < 0.05 compared to the respective control.

TrxR is an enzyme involved in the redox balance. Therefore, its inhibition causes a pro-oxidant state that leads to cancer cells’ death. Gold complexes may inactivate this protein due to their intrinsic affinity towards the selenocysteine residues of the enzyme [77,78]. In order to determine whether our complexes have this protein as a putative target, in vitro studies were carried out by incubating the protein with complexes **1** and **8** at their IC_50_ values. The results indicate that both compounds were capable of interacting in vitro with this enzyme, causing a significant decrease in its activity of around 80% (Figure 6A). The TrxR inhibitor, auranofin, was used as a reference.

Finally, the ability to inhibit CA IX activity was evaluated in order to confirm whether the new derivatives could behave as multi-target drugs. The fact that these dithiocarbamate gold(I) complexes are carrying benzenesulfonamide units suggests that the carbonic anhydrase enzyme could be an additional target, as has been previously observed in other compounds with benzenesulfonamide units [79,80]. We have chosen the CA IX isoform since it is more significantly expressed in tumor tissues. The results, shown in Figure 6B, indicate that complexes **1** and **8** produce a significant reduction in the esterase activity of CA at a concentration of 30 μM, with the former being more effective than the latter (48 and 31%, respectively). Furthemore, when indisulam, a specific inhibitor of this isoform, was used, its required concentration was twice (60 μM) that needed to achieve a similar effect corresponding to [Au(S_2_CNHSO_2_-p-Me-C_6_H_4_)(IMePropargyl)] (**8**). Thus, both compounds are capable of inhibiting the CA IX isoform at lower concentrations than the reference inhibitor, indisulam. The inhibition of this enzyme would impede the regulation of the pH, causing the death of the cells due to the acidity of the medium and hypoxic conditions [81]. 

With these results, we can conclude that both complexes could act as multi-target drugs by inhibiting the activity of both thioredoxine reductase and carbonic anhydrase IX enzymes.

#### 3.2.5. Effect of Gold(I) Complexes on Intracellular ROS

**Figure 7:**
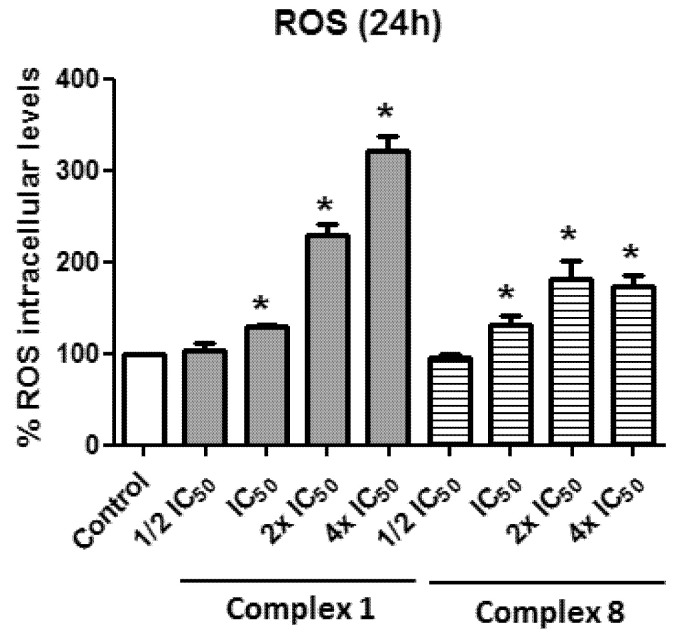
Measurement of the intracellular levels of ROS after the treatment of Caco-2 cells with complexes **1** and **8** at different concentrations for 24 h. * *p* < 0.05 compared to the control.

Another aspect to be taken into account in the search for new agents for cancer treatment is the ROS level alteration in tumor cells. Therefore, the use of drugs that induce the generation of these oxygen species is very useful, as cancer cells are much more susceptible to them [82,83]. As shown in Figure 7and Figure S28, these two metal complexes [Au(S_2_CNHSO_2_C_6_H_5_)(PPh_3_)] (**1**) and [Au(S_2_CNHSO_2_-p-Me-C_6_H_4_)(IMePropargyl)] (**8**) produced a time- and concentration-dependent pro-oxidant effect. This fact is in agreement with its inhibitory effect found on the TrxR1 protein (Figure 6A). In fact, many examples of gold complexes have previously shown the ability to increase the ROS levels in cancerous cells besides the inhibition of thioredoxine reductase activity [58,60,84,85,86,87,88].

However, we cannot rule out the fact that these coompounds activate the p53 protein (Figure 5). The role of p53 as a central component of the stress response machinery is well established, and numerous forms of stress found during malignant transformation can lead to the activation of p53 [89]. This protein can influence the redox response, being antioxidant or prooxidant depending on the cell stress. At low levels of stress, p53 acts to promote an antioxidant response, while high levels lead to a prooxidant response [83].

## 4. Conclusions

Ten new dithiocarbamate gold(I) complexes derived from benzenesulfonamide with phosphines or carbenes as ancillary ligands were isolated as stable products. They exert a cytotoxic effect on the Caco-2 line, which depends on the nature of the ligand, with the dithiocarbamate derivatives with PPh_3_ being the most effective. The low toxicity of the complexes with the water-soluble phosphine TPPTS stands out, probably due to their hydrophilic nature, which could hinder their uptake through the membrane. The complexes [Au(S_2_CNHSO_2_C_6_H_5_)(PPh_3_)] (**1**) and [Au(S_2_CNHSO_2_-p-Me-C_6_H_4_)(IMePropargyl)] (**8**) trigger intrinsic apoptosis in Caco-2 cells by modifying the mitochondrial potential and activating caspase 3. Both complexes generate cellular stress that causes the activation of the p53 protein, prompting cell cycle arrest in the G1 phase. The inhibition of thioredoxin reductase causes a pro-oxidant effect without ruling out a possible pro-oxidant action of protein p53. Likewise, the activity of carbonic anhydrase (CA IX isoform) is compromised, which indicates that both complexes can be considered as multi-target drugs. Besides this, [Au(S_2_CNHSO_2_C_6_H_5_)(PPh_3_)] (**1**) displays higher anticancer activity, time independently, than its carbene counterpart ([Au(S_2_CNHSO_2_-p-Me-C_6_H_4_)(IMePropargyl)] (**8**)), which required a period of activation. 

## Figures and Tables

**Table 1:** IC_50_ values obtained after treatment for 72 h with dithiocarbamate gold(I) complexes derived from sulfonamides on Caco-2 cells, along with the distribution coefficients (log P_7.4_).

Complex	IC_50_ (μM)	Log P_7.4_
[Au(S_2_CNHSO_2_C_6_H_5_)(PPh_3_)] (**1**)	1.14 ± 0.25	0.75
[Au(S_2_CNHSO_2_-p-Me-C_6_H_4_)(PPh_3_)] (**2**)	1.25 ± 0.35	0.71
[Au(S_2_CNHSO_2_-p-Cl-C_6_H_4_)(PPh_3_)] (**3**)	1.70 ± 0.15	0.91
[Au(S_2_CNHSO_2_C_6_H_5_)(TPPTS)] (**4**)	46.71 ± 5.61	−0.40
[Au(S_2_CNHSO_2_-p-Me-C_6_H_4_)(TPPTS)] (**5**)	31.81 ± 6.05	−0.78
[Au(S_2_CNHSO_2_-p-Cl-C_6_H_4_)(TPPTS)] (**6**)	33.68 ± 2.74	−0.83
[Au(S_2_CNHSO_2_C_6_H_5_)(IMePropargyl)] (**7**)	33.27 ± 5.01	0.35
[Au(S_2_CNHSO_2_-p-Me-C_6_H_4_)(IMePropargyl)] (**8**)	7.22 ± 0.38	−0.13
[Au(S_2_CNHSO_2_C_6_H_5_)(IBnPropargyl)] (**9**)	8.56 ± 1.68	0.42
[Au(S_2_CNHSO_2_-p-Me-C_6_H_4_)(IBnPropargyl)] (**10**)	13.15 ± 3.34	0.36

**Table 2:** IC_50_ values and the corresponding selectivity index obtained after treatment for 72 h with dithiocarbamate gold(I) complexes derived from sulfonamides on differentiated Caco-2 cells.

Complex	IC_50_ (μM)	Selectivity Index
[Au(S_2_CNHSO_2_C_6_H_5_)(PPh_3_)] (**1**)	12.72 ± 0.47	11.2
[Au(S_2_CNHSO_2_-p-Cl-C_6_H_4_)(PPh_3_)] (**3**)	14.28 ± 0.19	8.05
[Au(S_2_CNHSO_2_-p-Me-C_6_H_4_)(IMePropargyl)] (**8**)	46.62 ± 3.26	6.46
[Au(S_2_CNHSO_2_C_6_H_5_)(IBnPropargyl)] (**9**)	19.54 ± 2.64	2.28

**Table 3:** IC_50_ values obtained after treating Caco-2 cells with complexes **1**, **3**, **8** and **9** at incubation times of 24, 48 and 72 h.

Complex	IC_50_ 24 h (μM)	IC_50_ 48 h (μM)	IC_50_ 72 h (μM)
[Au(S_2_CNHSO_2_C_6_H_5_)(PPh_3_)] (**1**)	1.92 ± 0.13	1.38 ± 0.21	1.14 ± 0.25
[Au(S_2_CNHSO_2_-p-Cl-C_6_H_4_)(PPh_3_)] (**3**)	2.48 ± 0.14	1.51 ± 0.10	1.70 ± 0.15
[Au(S_2_CNHSO_2_-p-Me-C_6_H_4_)(IMePropargyl)] (**8**)	>50	37.41 ± 7.48	7.22 ± 0.38
[Au(S_2_CNHSO_2_C_6_H_5_)(IBnPropargyl)] (**9**)	31.25 ± 6.85	15.48 ± 1.89	8.56 ± 1.68

## Data Availability

The data are contained within the article or Supplementary Material.

## Supplementary Materials

The following supporting information can be downloaded at https://www.mdpi.com/article/10.3390/biomedicines10061437/s1. Figures S1–S26: NMR spectra of the complexes; Figure S27: UV–Vis spectra of the complexes; Figure S28: Measurement of the intracellular levels of ROS.
