# Supplementary material for: Sulfonamide-Derived Dithiocarbamate Gold(I) Complexes Induce the Apoptosis of Colon Cancer Cells by the Activation of Caspase 3 and Redox Imbalance
Source: Biomedicines. 2022 Jun 17;10(6):1437. doi: 10.3390/biomedicines10061437 (PMC9221018; doi:10.3390/biomedicines10061437)
Supplement: Supplementary file 1 [file biomedicines-10-01437-s001.zip › biomedicines-1756472-supplementary.pdf]

Therapeutic applications of Dithiocarbamate Sulfonamide-Derived Dithiocarbamate Gold(I) Complexes Induce Apoptosis of Colon Cancer Cells by Activation of Caspase 3 and Redox Imbalance

Javier Quero[1,2], José Carlos Royo[1,2], Beatrice Fodor[2], M. Concepción Gimeno[2], Jesús Osada[3,4], M. Jesús Rodríguez-Yoldi,[1,4*] and Elena Cerrada,[2*]

[1] Departamento de Farmacología y Fisiología y Medicina Legal y Forense. Unidad de Fisiología, Universidad de Zaragoza, 50013, Zaragoza, Spain, E-mail: mjrodyol@unizar.es (M.R.-Y.), 558203@unizar.es (J.Q.) and 720204@unizar.es (J.C.R.)

[2] Departamento de Química Inorgánica, Instituto de Síntesis Química y Catálisis Homogénea-ISQCH, Universidad de Zaragoza-C.S.I.C., 50009 Zaragoza, Spain. E-mail: ecerrada@unizar.es (E.C.); and gimeno@unizar.es (M.C.G.) and 822220@unizar.es (B.F)

[3] Departamento de Bioquímica y Biología Molecular y Celular. Universidad de Zaragoza, 50013, Zaragoza, Spain. E-mail: josada@unizar.es (J.O.)

[4] CIBERobn, ISCIII, IIS Aragón, IA2 (Spain).

Correspondence: mjrodyol@unizar.es (M.J.R.-Y.); Tel: +34-976-761649 and ecerrada@unizar.es (E.C.); Tel. +34-976-761181

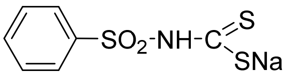

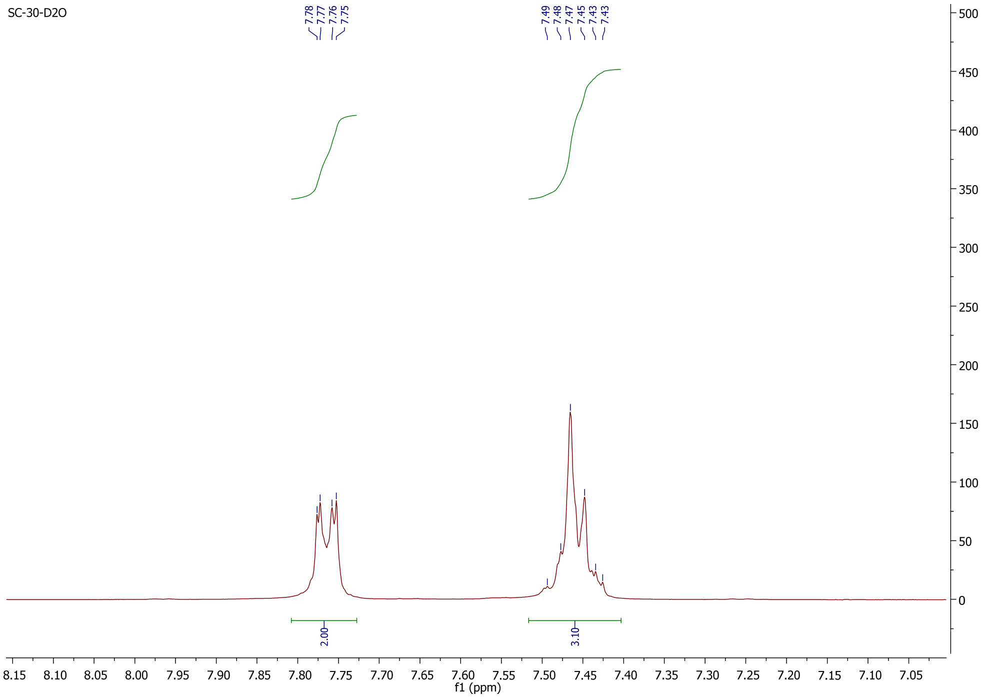

Figure S1. ^{1}H NMR spectrum of compound **L1** in D_2O.

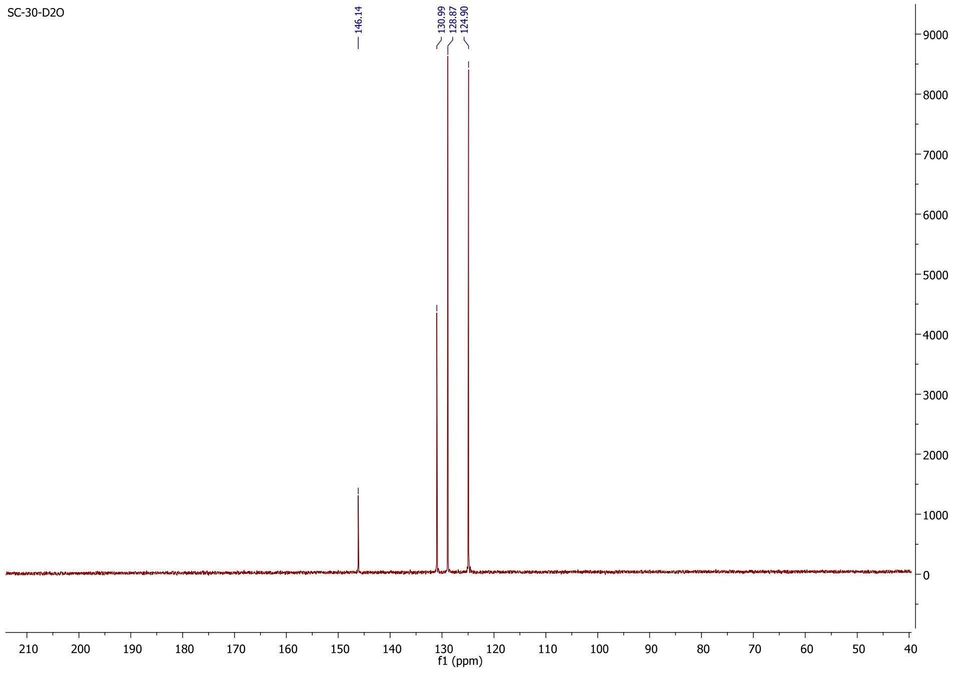

Figure S2. $^{13}C\{^{1}H\}$ NMR spectrum of compound **L1** in in D_2O.

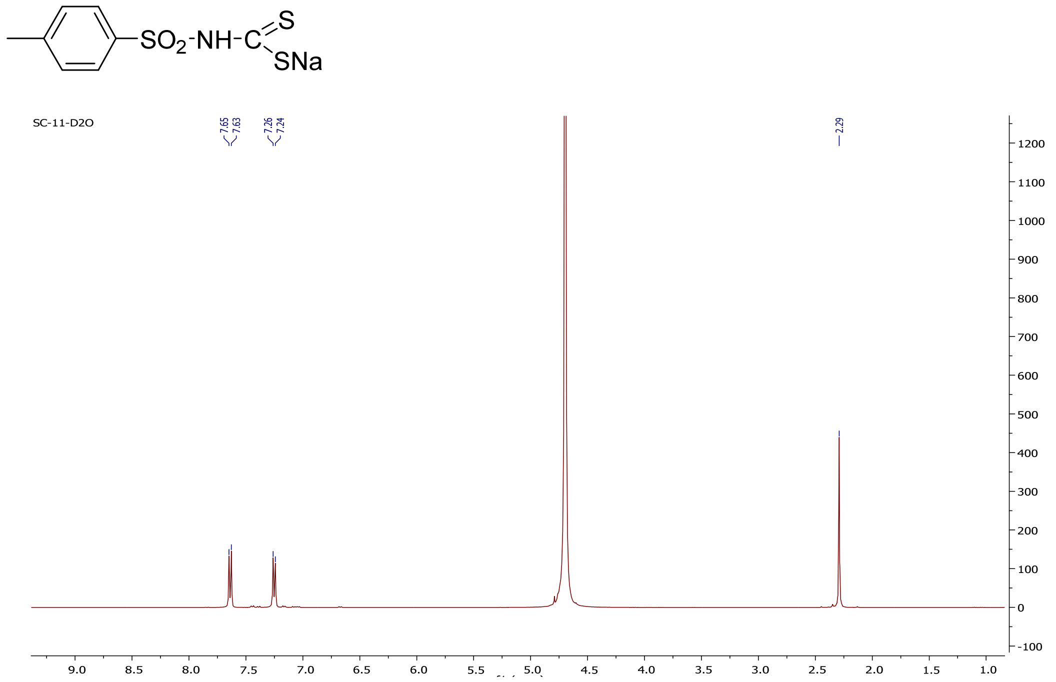

Figure S3. ^{1}H NMR spectrum of compound **L2** in D_2O.

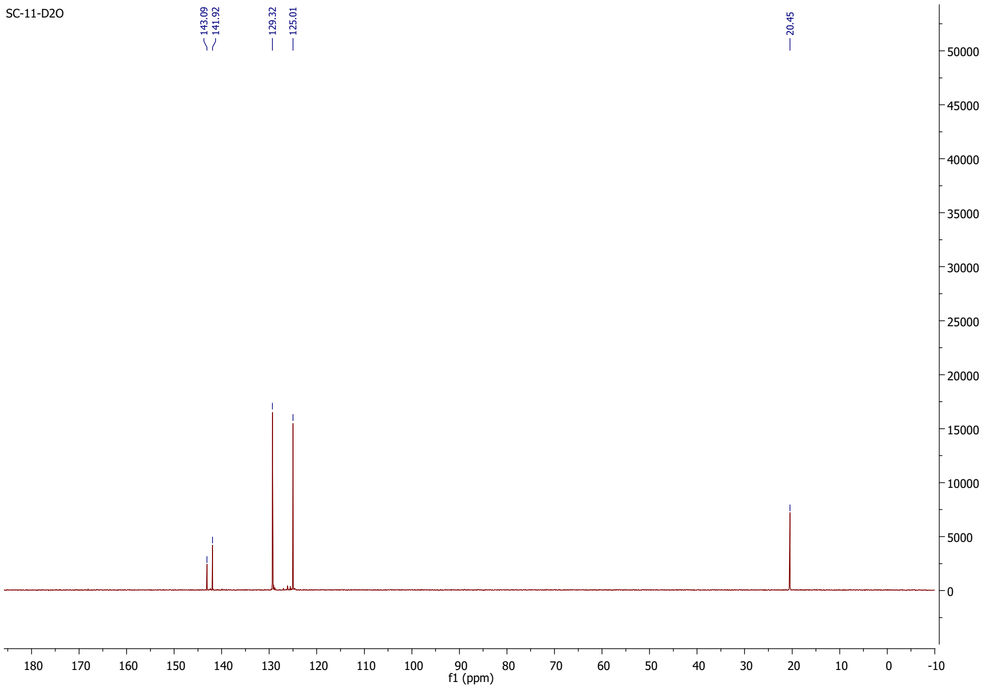

Figure S4. $^{13}C\{^{1}H\}$ NMR spectrum of compound **L2** in in D_2O.

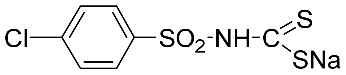

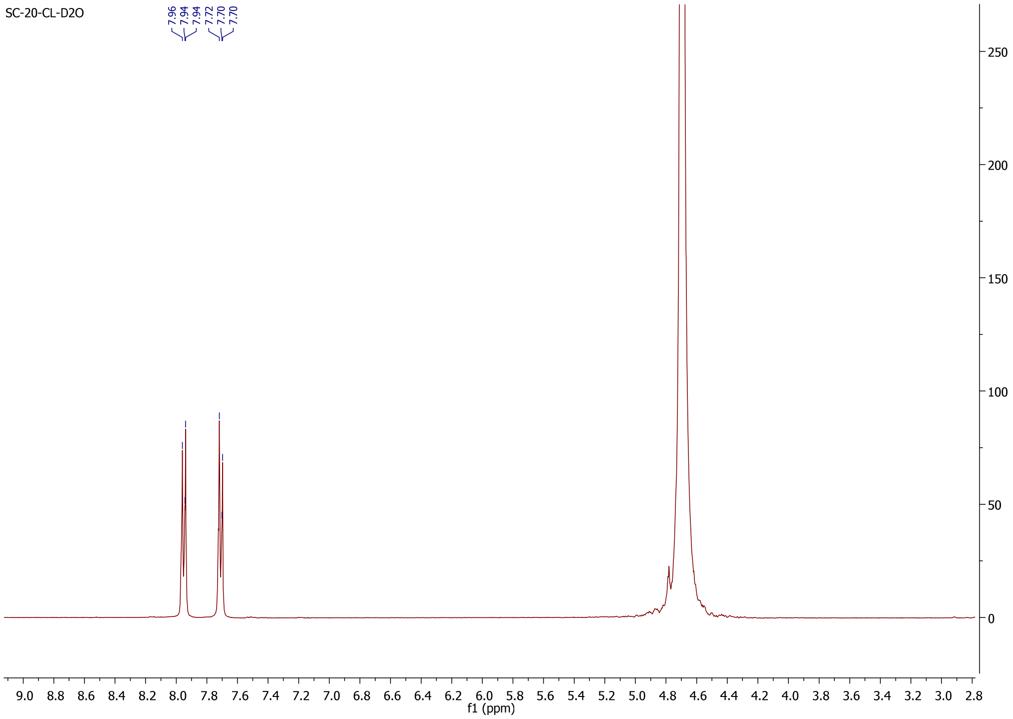

Figure S5. ^{1}H NMR spectrum of compound **L3** in dmso-d6.

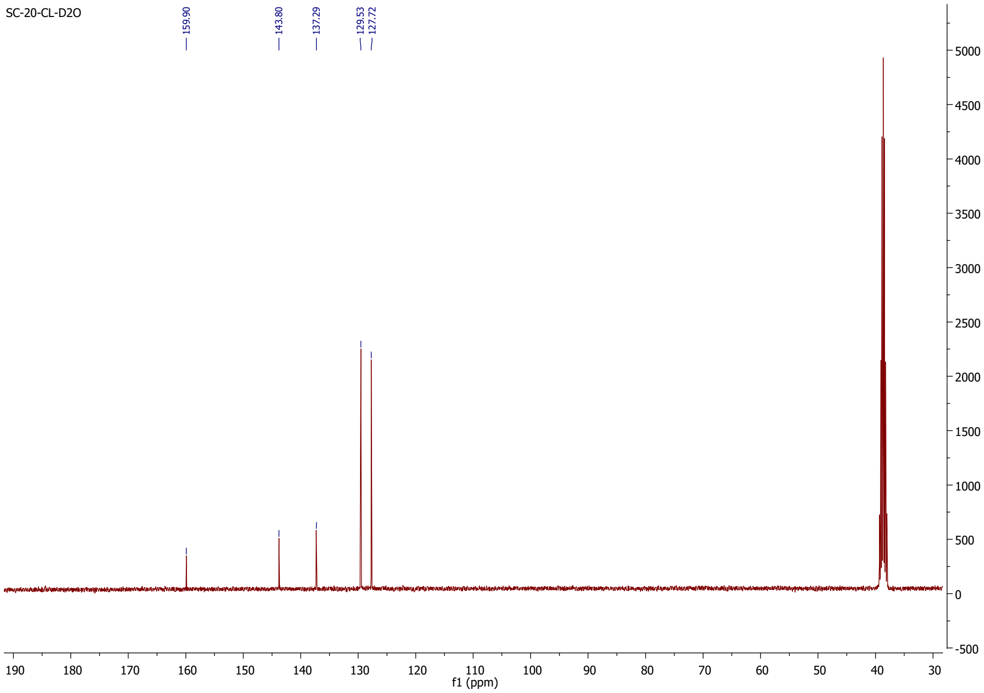

Figure S6. $^{13}C\{^{1}H\}$ NMR spectrum of compound **L3** in dmso-d6.

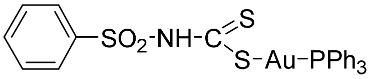

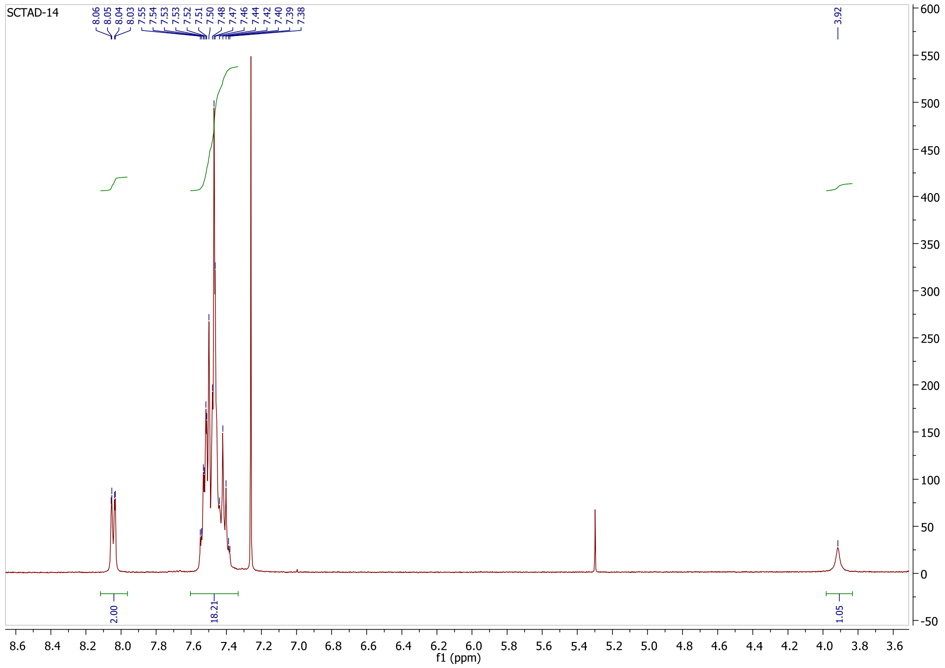

Figure S7. ^{1}H NMR spectrum of compound **1** in $CDCl_3$.

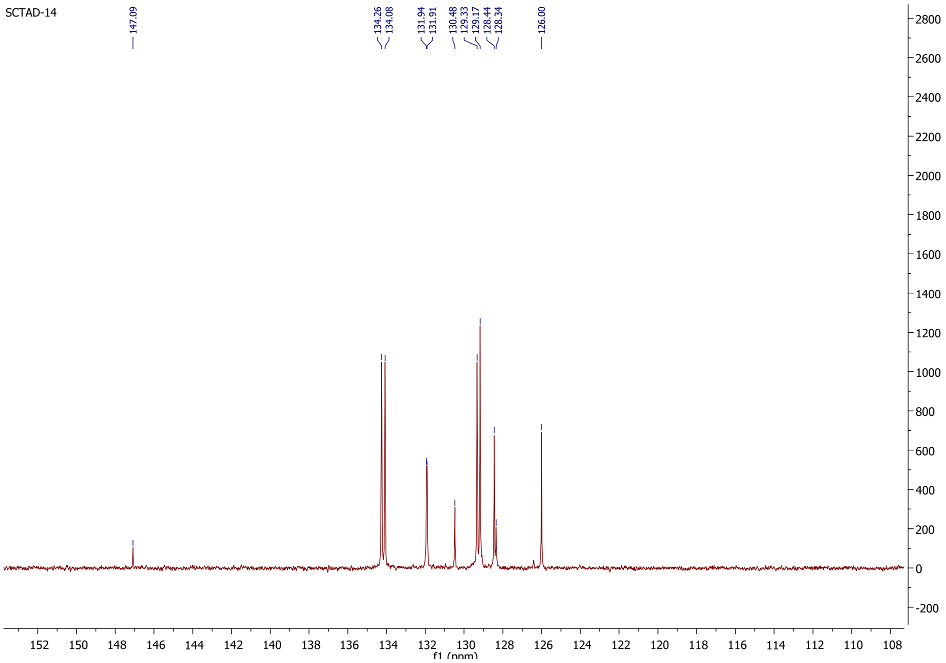

Figure S8. $^{13}C\{^{1}H\}$ NMR spectrum of compound **1** in $CDCl_3$.

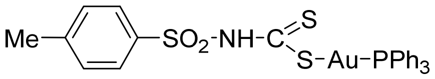

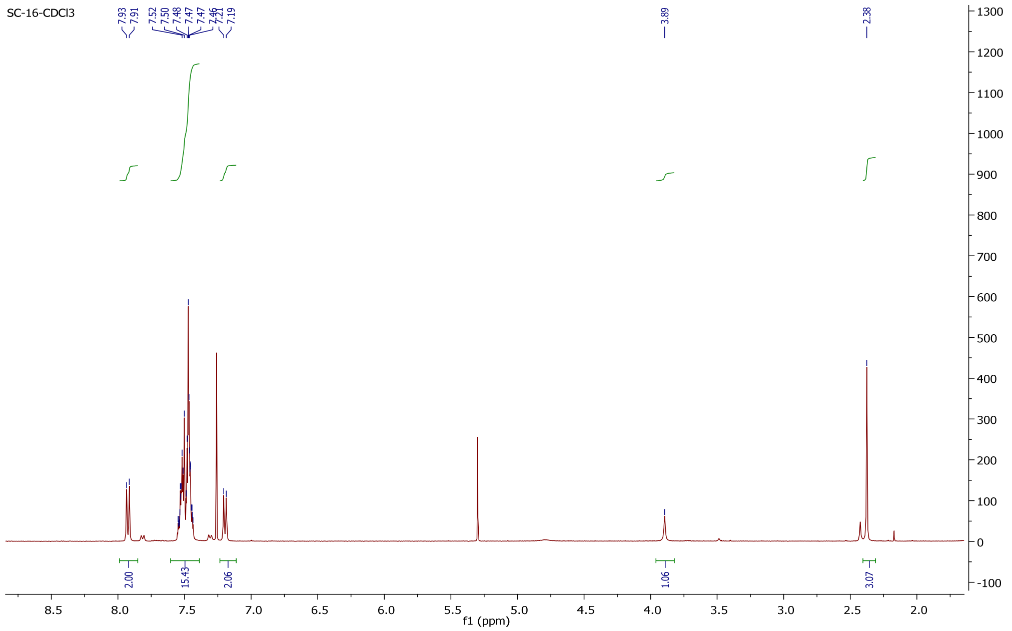

Figure S9. ^{1}H NMR spectrum of compound **2** in $CDCl_3$.

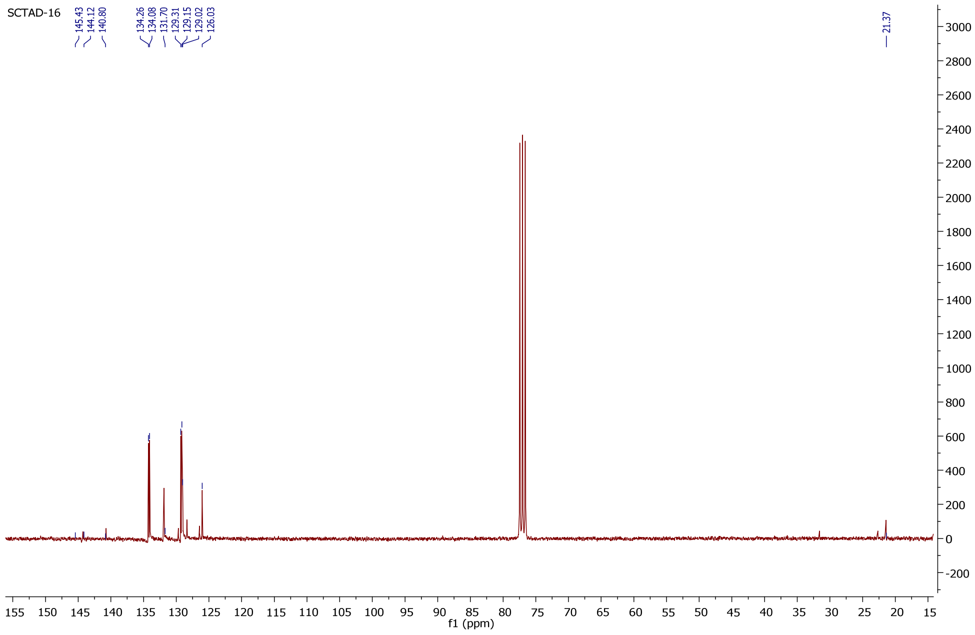

Figure S10. $^{13}C\{^{1}H\}$ NMR spectrum of compound **2** in $CDCl_3$.

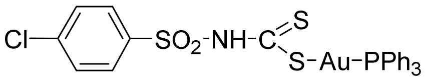

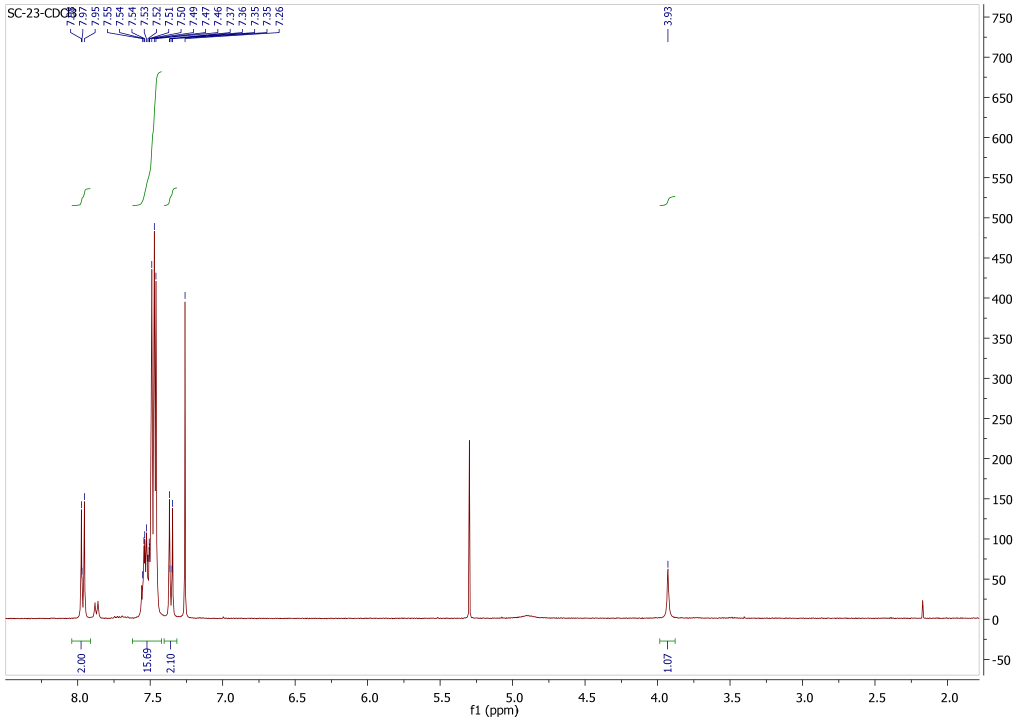

Figure S11. ^{1}H NMR spectrum of compound **3** in $CDCl_3$.

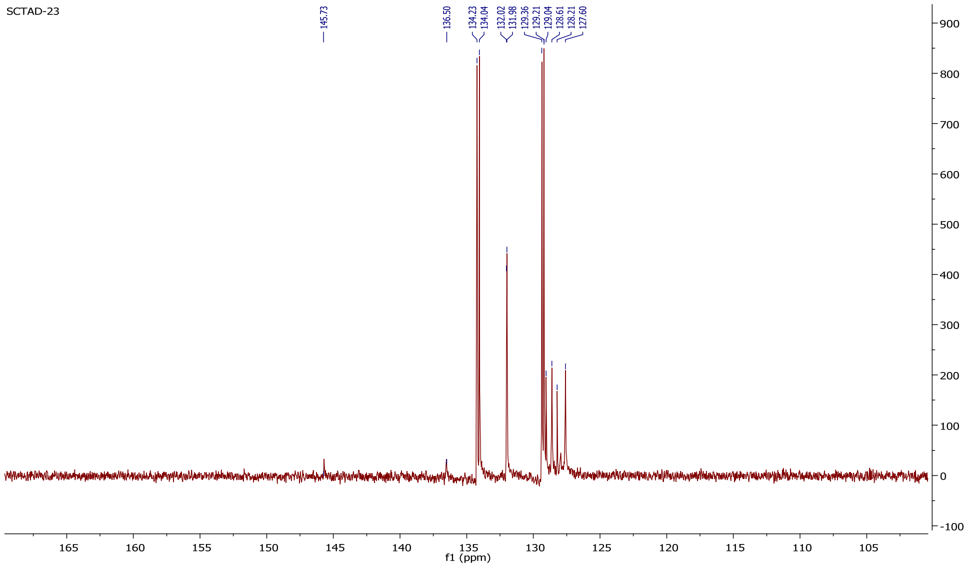

Figure S12. $^{13}C\{^{1}H\}$ NMR spectrum of compound **3** in $CDCl_3$.

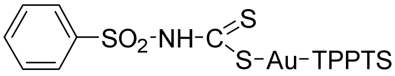

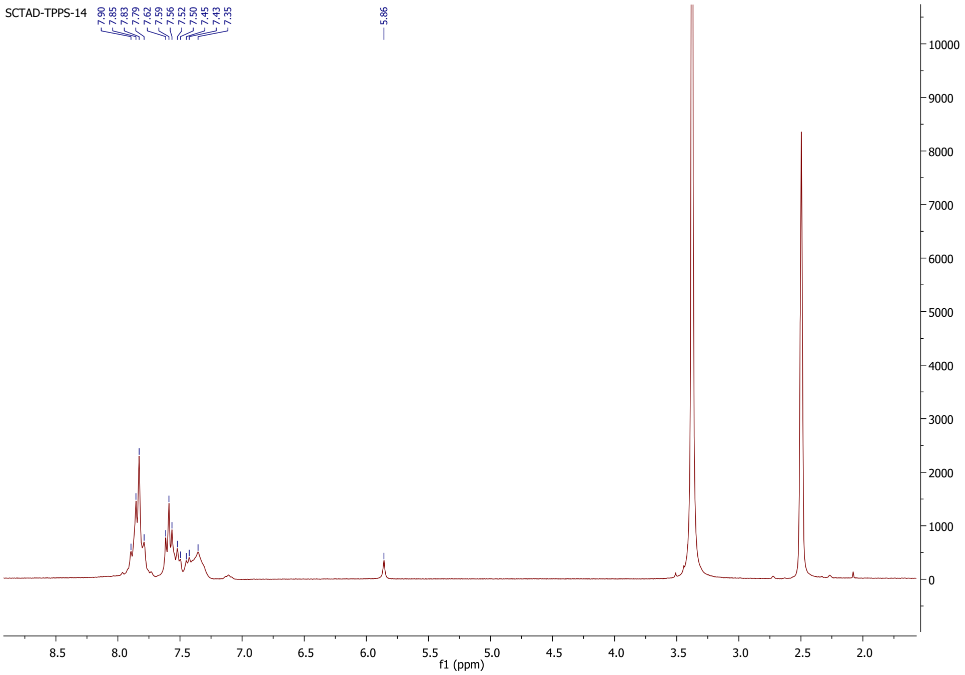

Figure S13. ^{1}H NMR spectrum of compound **4** in dmso-d_6.

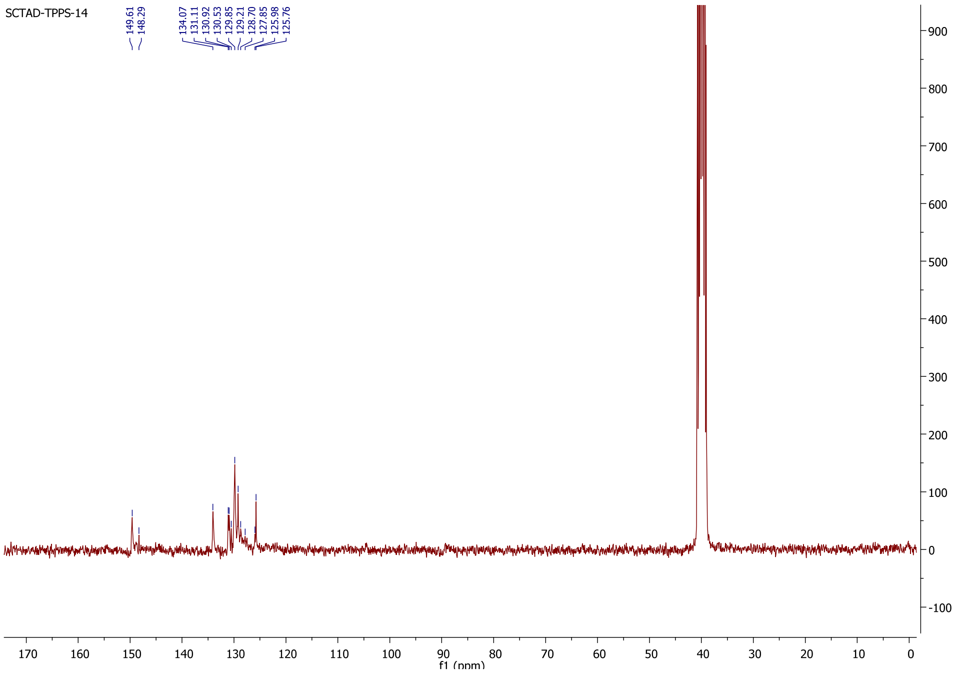

Figure S14. ^{13}C{^{1}H} NMR spectrum of compound **4** in dmso-d_6.

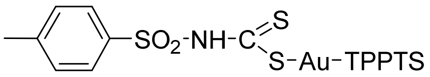

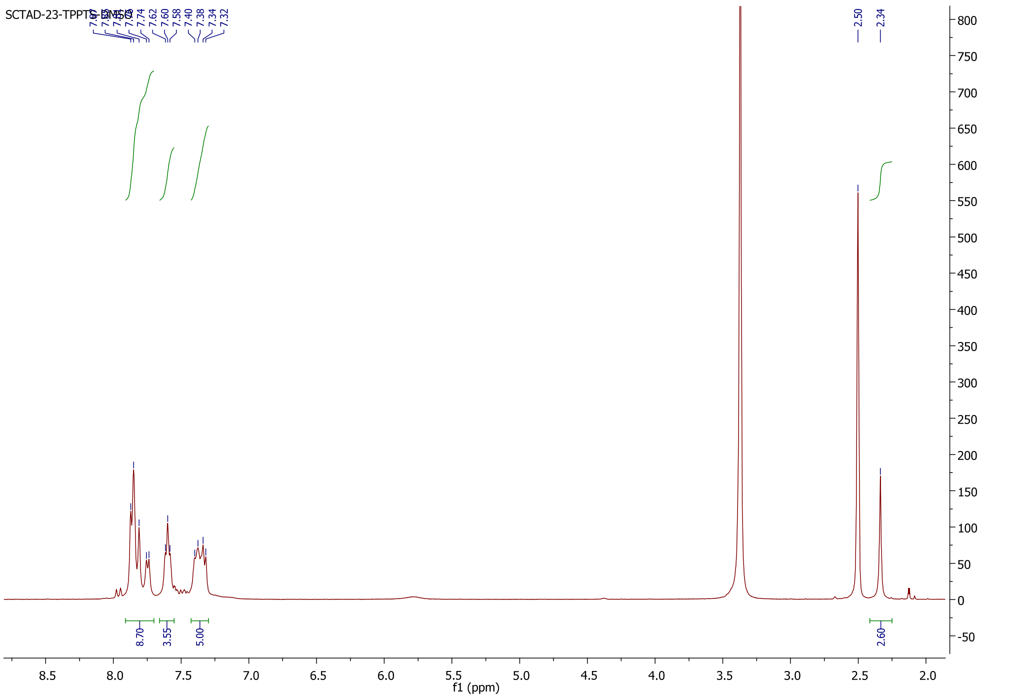

Figure S15. ^{1}H NMR spectrum of compound **5** in dmso-d_6.

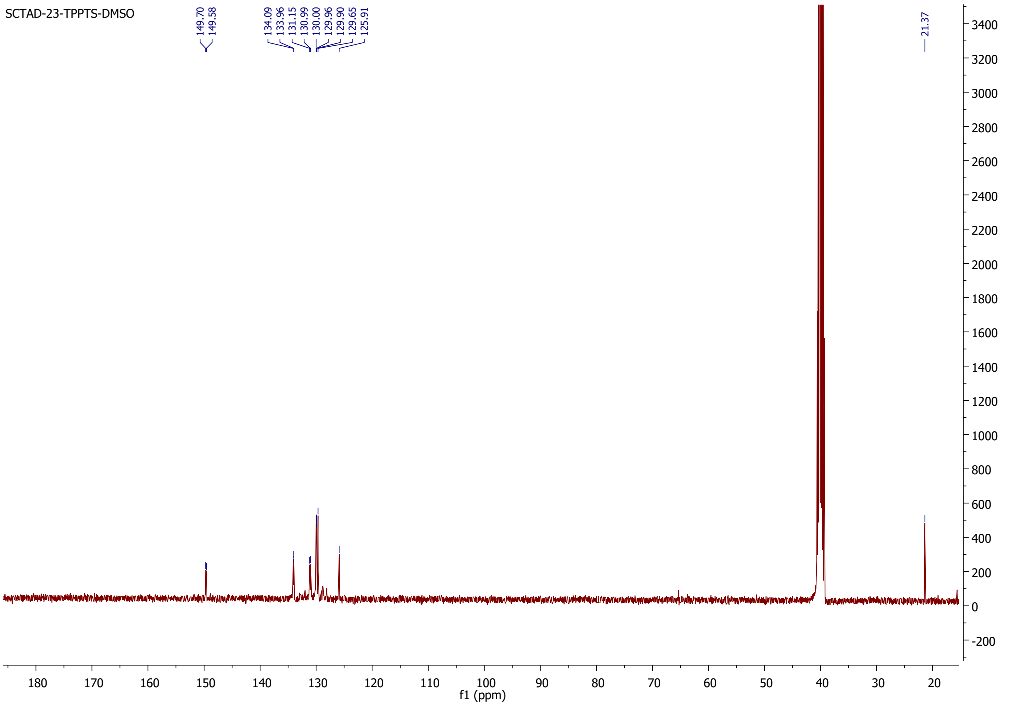

Figure S16. ^{13}C{^{1}H} NMR spectrum of compound **5** in dmso-d_6.

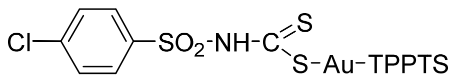

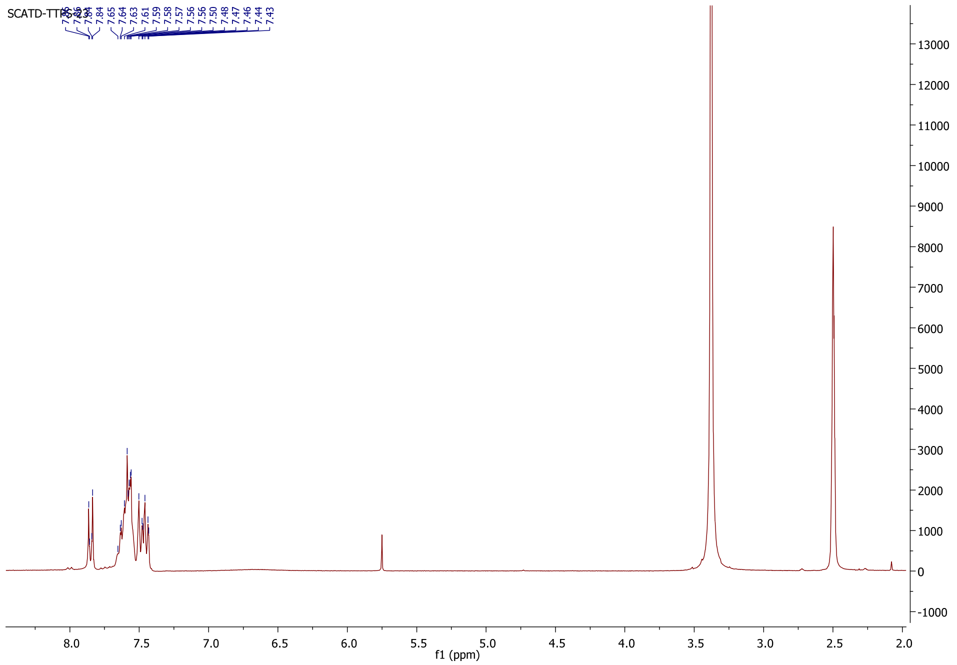

Figure S17. ^{1}H NMR spectrum of compound **6** in dmso-d_6.

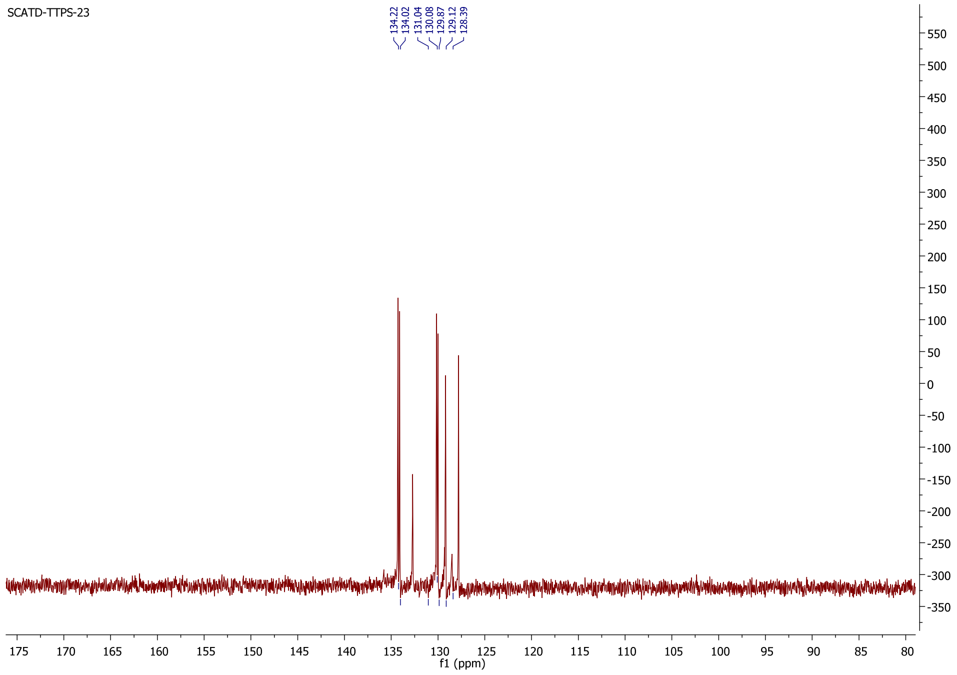

Figure S18. ^{13}C{^{1}H} NMR spectrum of compound **6** in dmso-d_6.

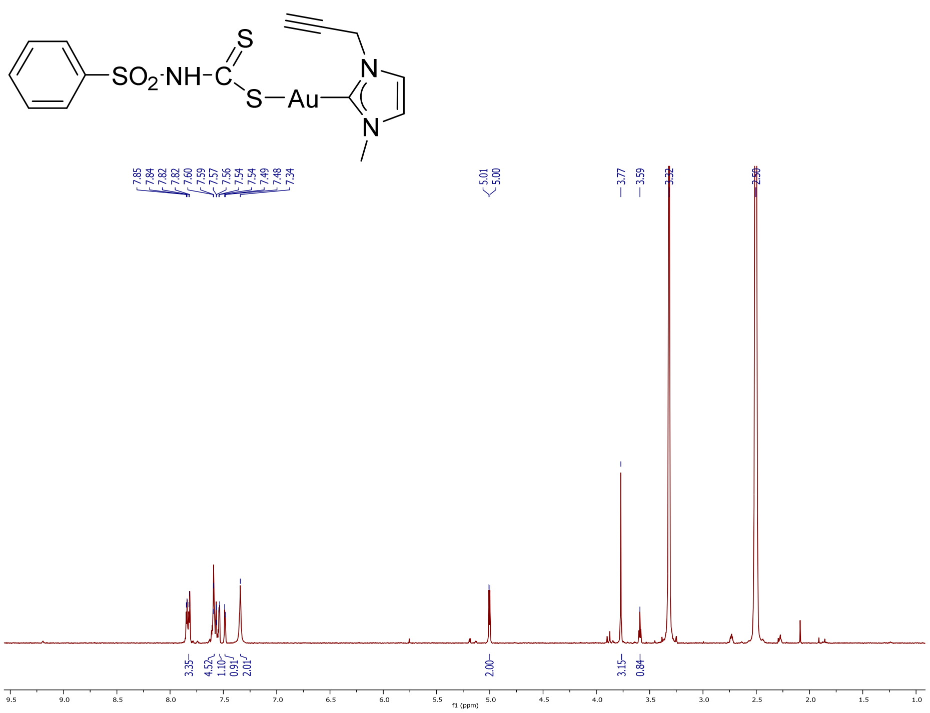

Figure S19. ^{1}H NMR spectrum of compound **7** in dmso-d_6.

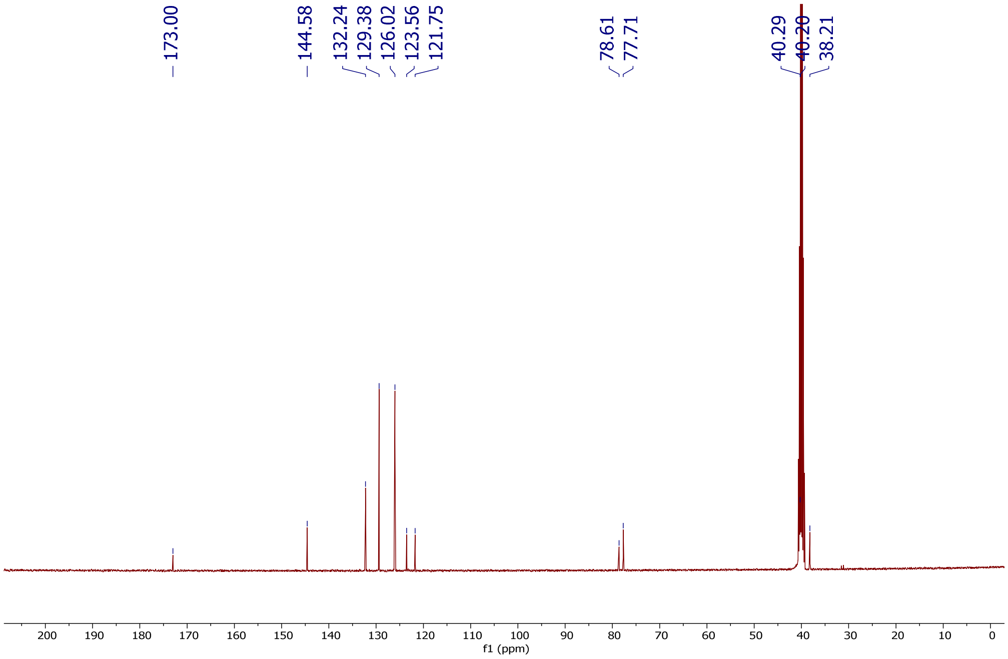

Figure S20. ^{13}C{^{1}H} NMR spectrum of compound **7** in dmso-d_6.

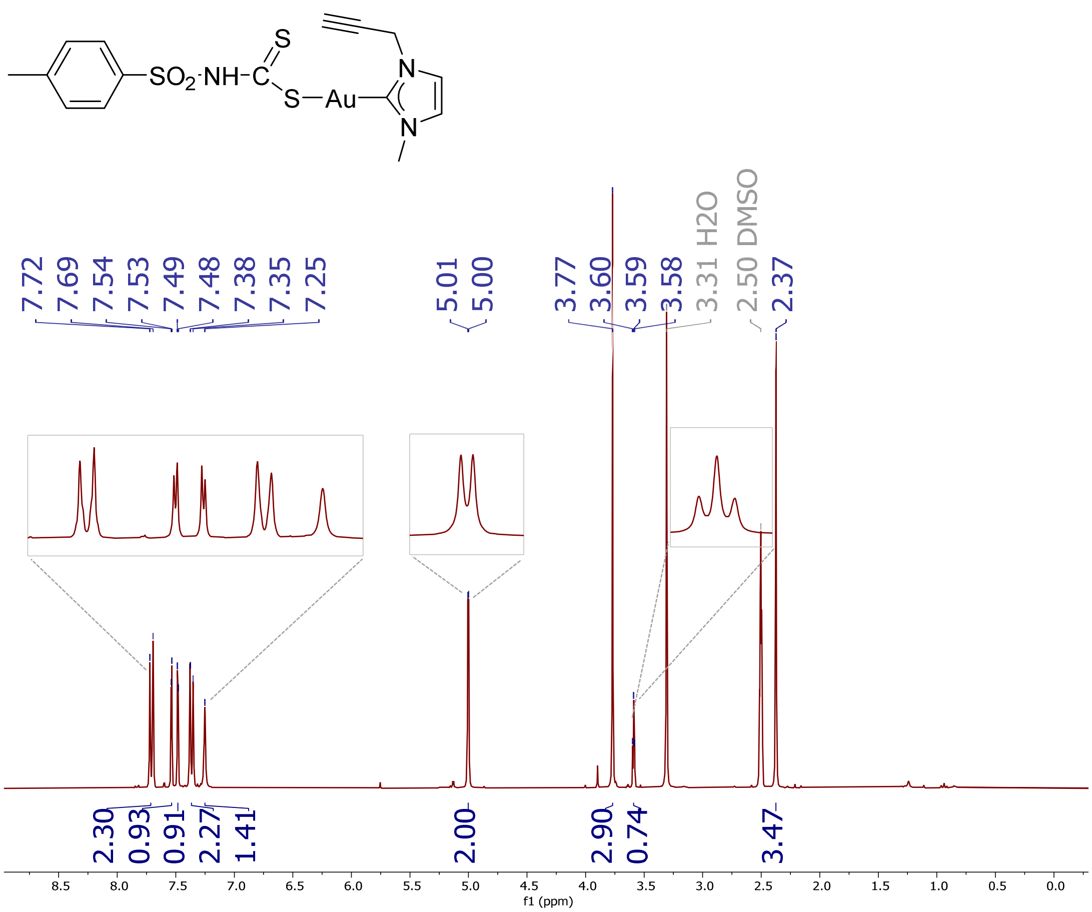

Figure S21. ^{1}H NMR spectrum of compound **8** in dmso-d_6.

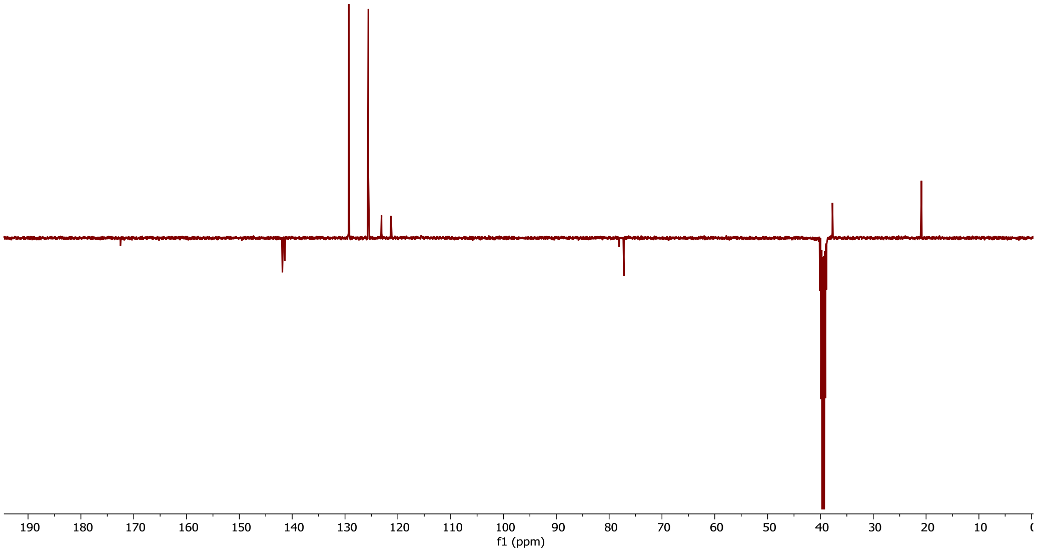

Figure S22. $^{13}C\{^{1}H\}$-apt NMR spectrum of compound **8** in dmso-d_6.

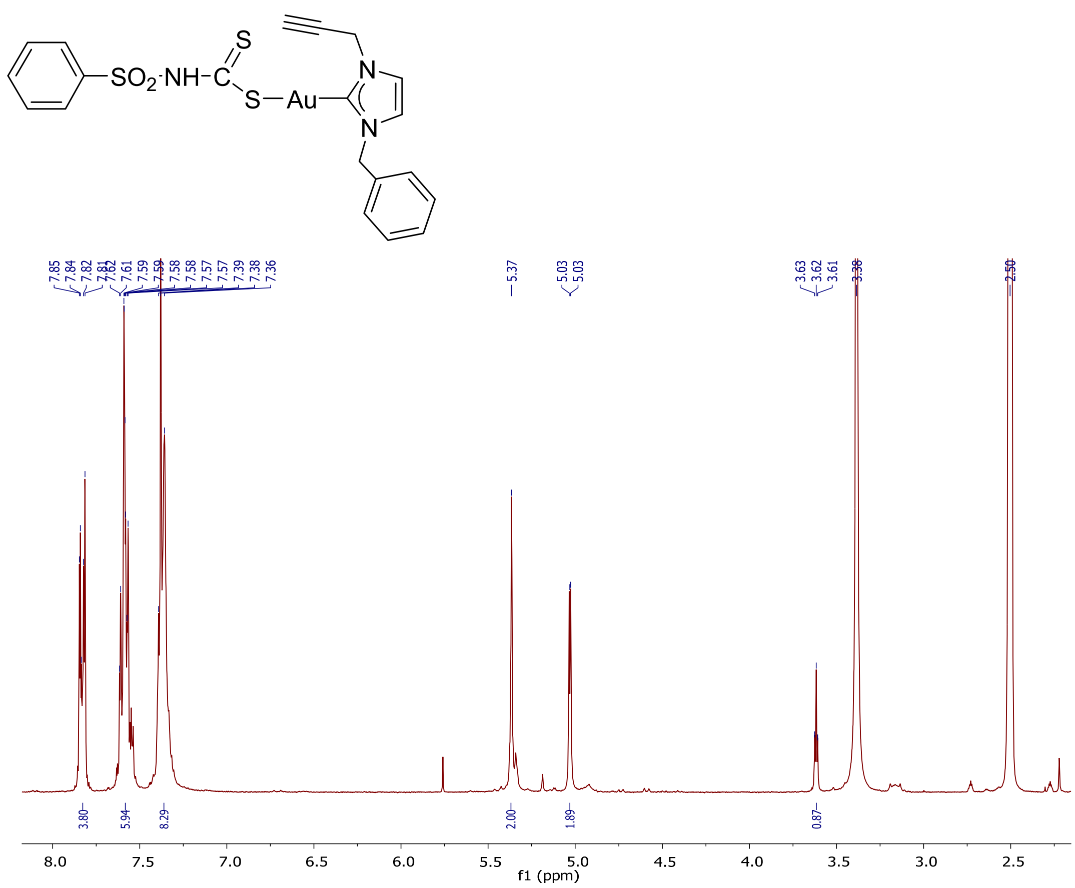

Figure S23. ^{1}H NMR spectrum of compound **9** in dmso-d_6.

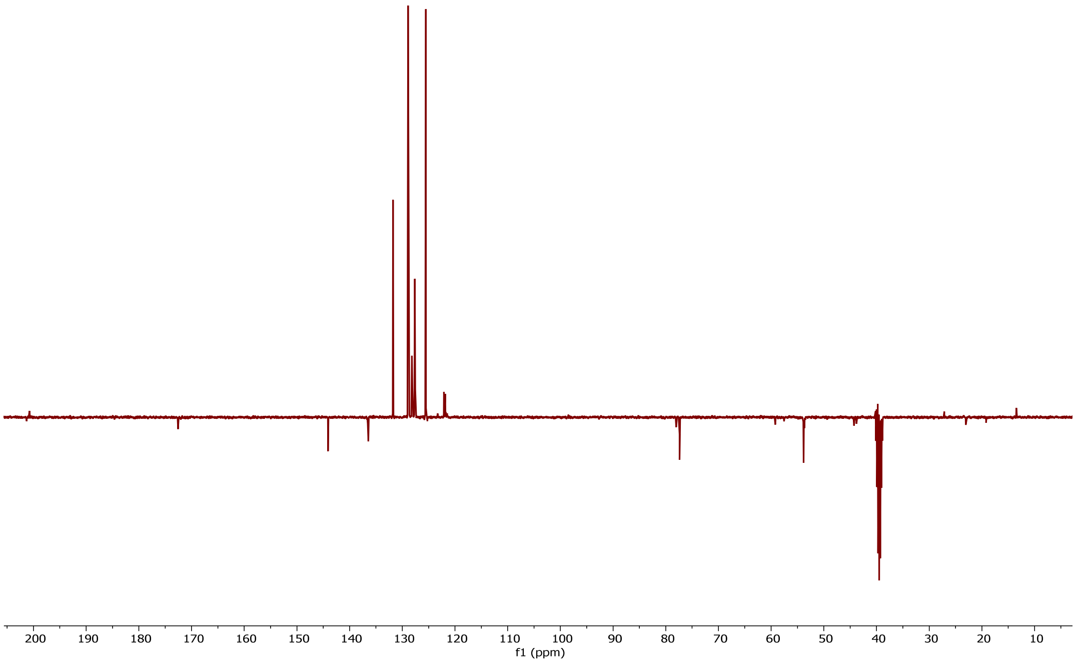

Figure S24. ^{13}C{^{1}H} NMR spectrum of compound **9** in dmso-d_6.

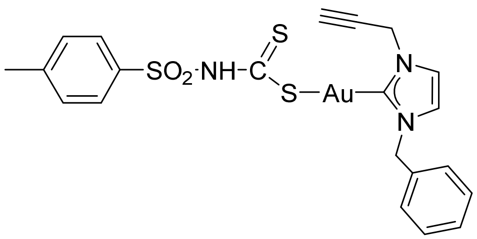

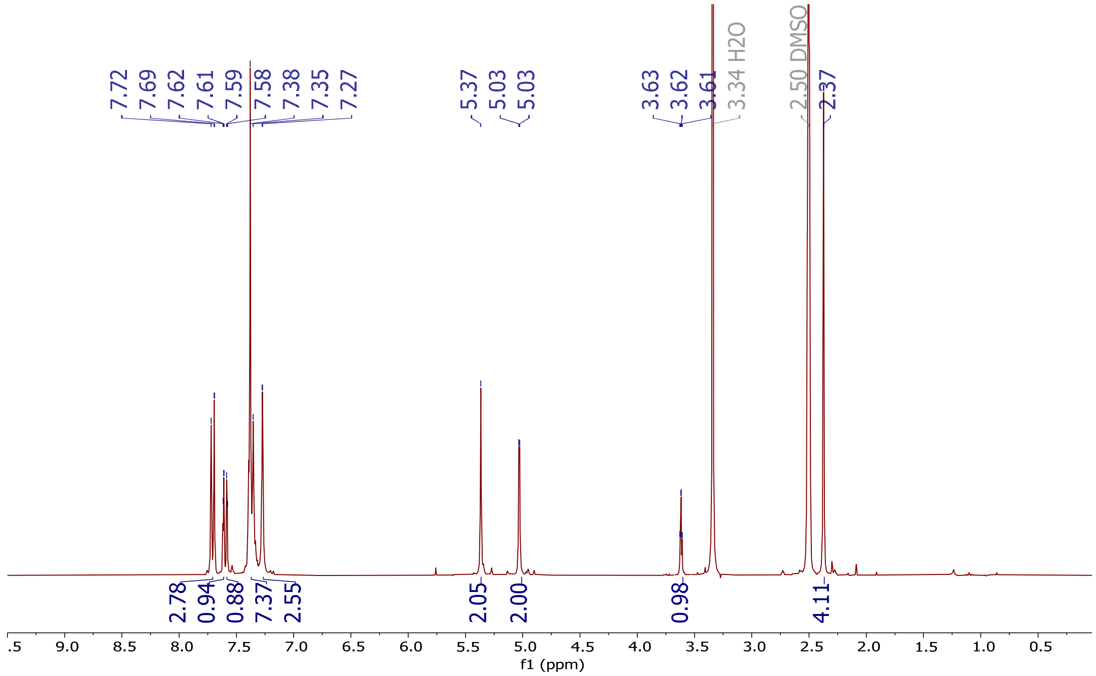

Figure S25. ^{1}H NMR spectrum of compound **10** in dmso-d_6.

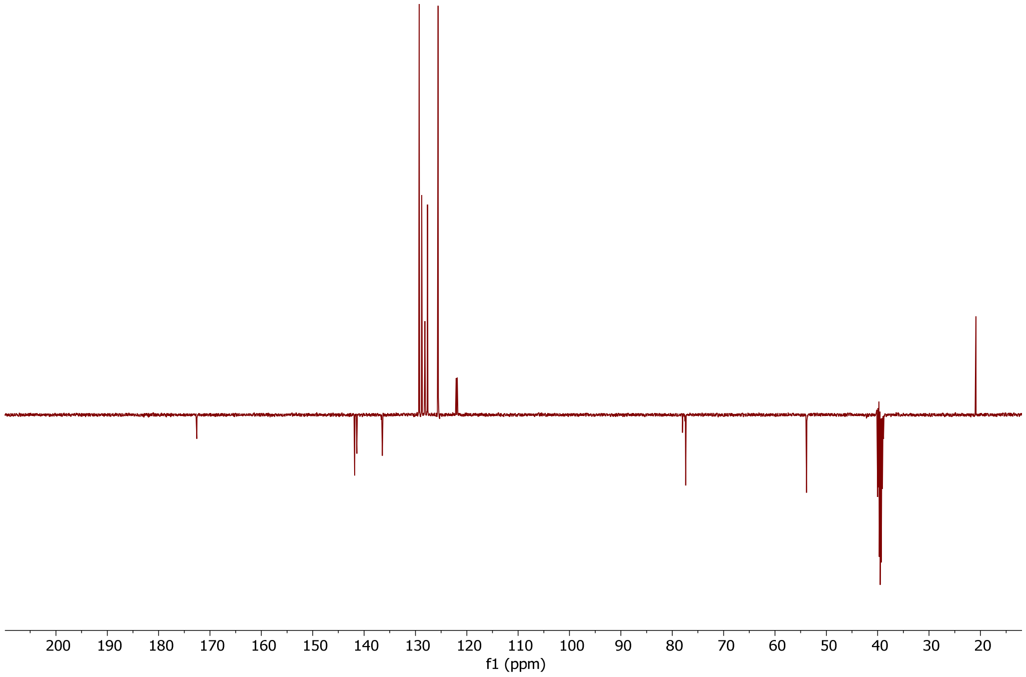

Figure S26. $^{13}C\{^{1}H\}$-apt NMR spectrum of compound **10** in dmso-d_6.

1

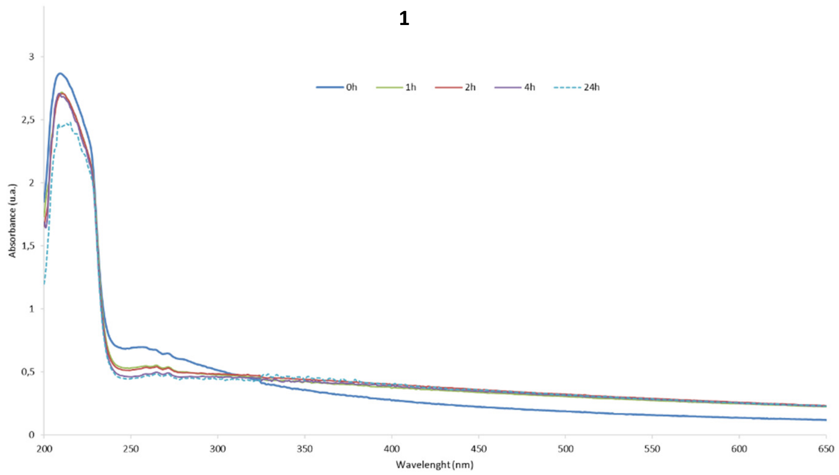
0h
1h
2h
4h
24h
Absorbance (u.a.)
Wavelenght (nm)
0
0,5
1
1,5
2
2,5
3
200
250
300
350
400
450
500
550
600
650

2

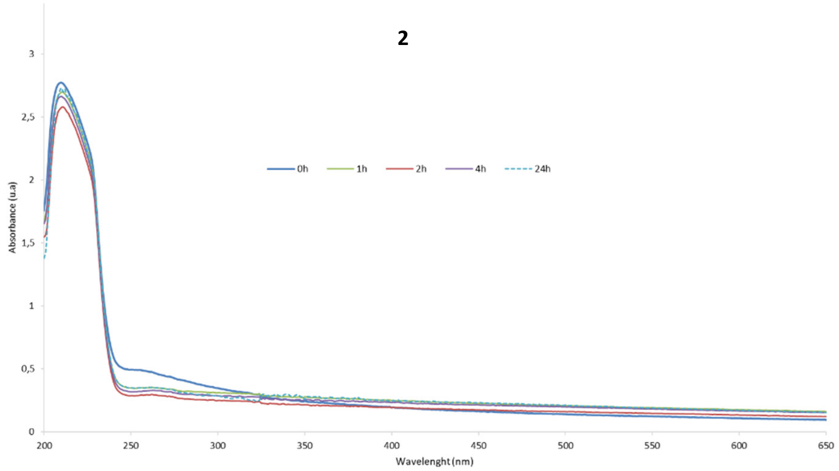
0h
1h
2h
4h
24h
Absorbance (u.a)
Wavelenght (nm)
0
0,5
1
1,5
2
2,5
3
200
250
300
350
400
450
500
550
600
650

3

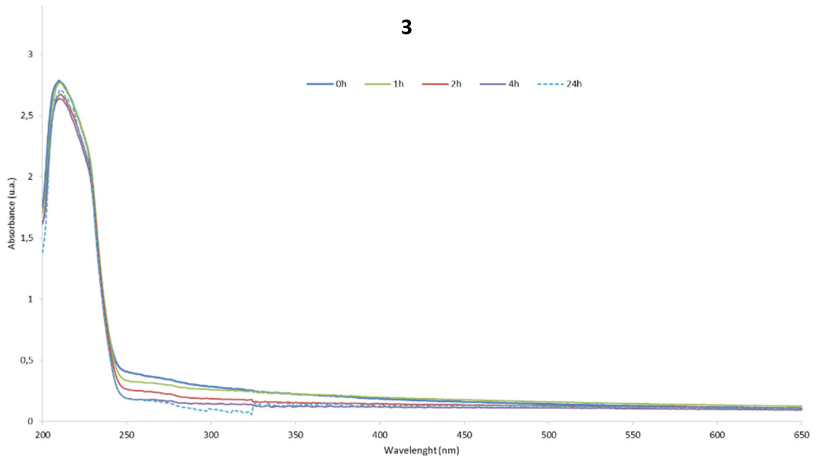
0h
1h
2h
4h
24h
Absorbance (u.a.)
Wavelenght (nm)
0
0,5
1
1,5
2
2,5
3
200
250
300
350
400
450
500
550
600
650

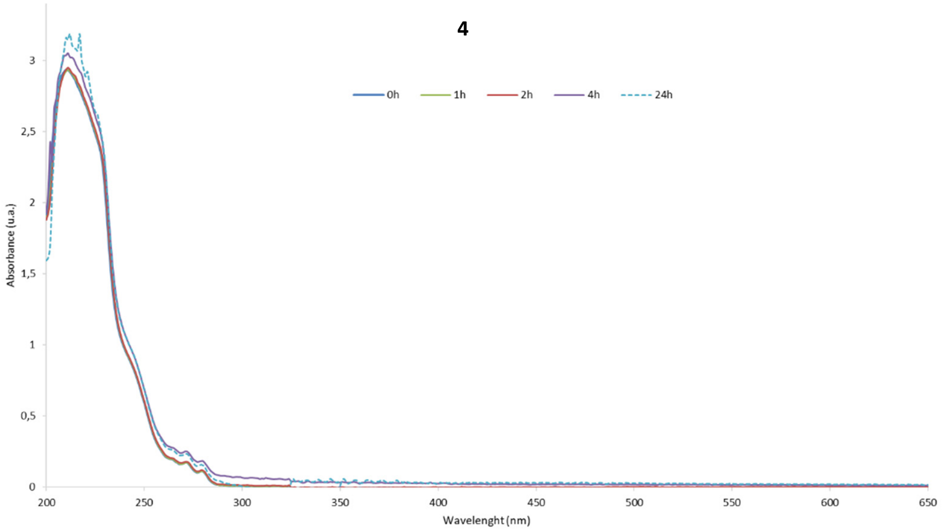
4
0h
1h
2h
4h
24h
Absorbance (u.a.)
0
0,5
1
1,5
2
2,5
3
Wavelenght (nm)
200
250
300
350
400
450
500
550
600
650

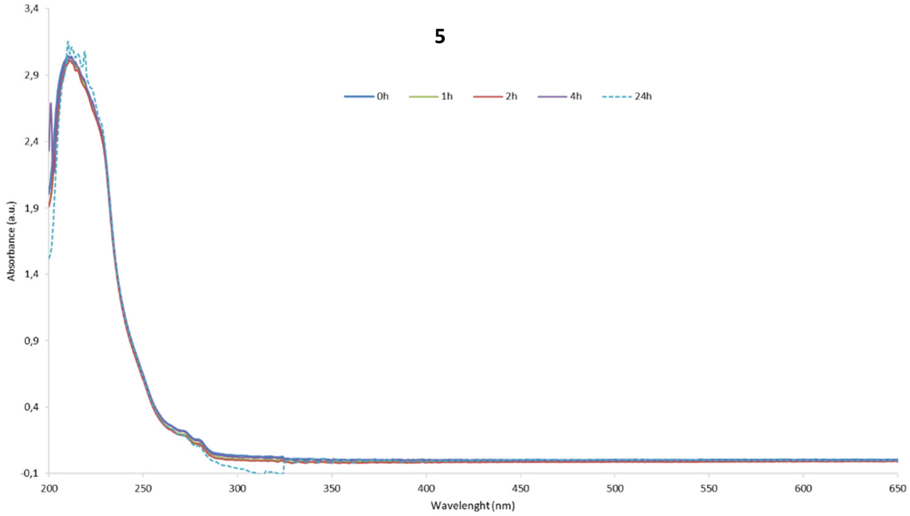
5
0h
1h
2h
4h
24h
Absorbance (a.u.)
-0,1
0,4
0,9
1,4
1,9
2,4
2,9
3,4
Wavelenght (nm)
200
250
300
350
400
450
500
550
600
650

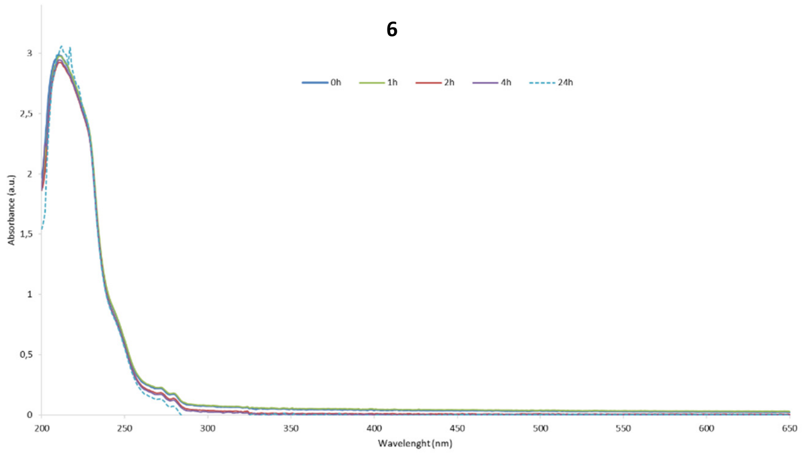
6
0h
1h
2h
4h
24h
Absorbance (a.u.)
0
0,5
1
1,5
2
2,5
3
Wavelenght (nm)
200
250
300
350
400
450
500
550
600
650

7

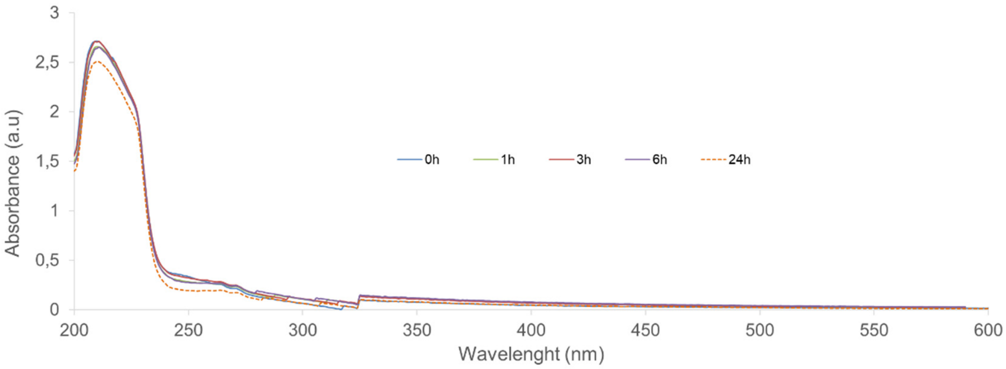

Absorbance (a.u)
Wavelenght (nm)
0h
1h
3h
6h
24h
0
0,5
1
1,5
2
2,5
3
200
250
300
350
400
450
500
550
600

8

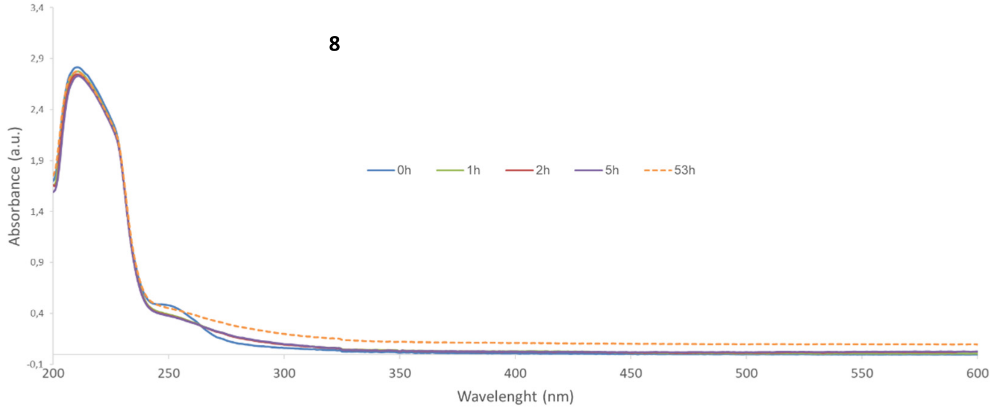

Absorbance (a.u.)
Wavelenght (nm)
0h
1h
2h
5h
53h
-0,1
0,4
0,9
1,4
1,9
2,4
2,9
3,4
200
250
300
350
400
450
500
550
600

9

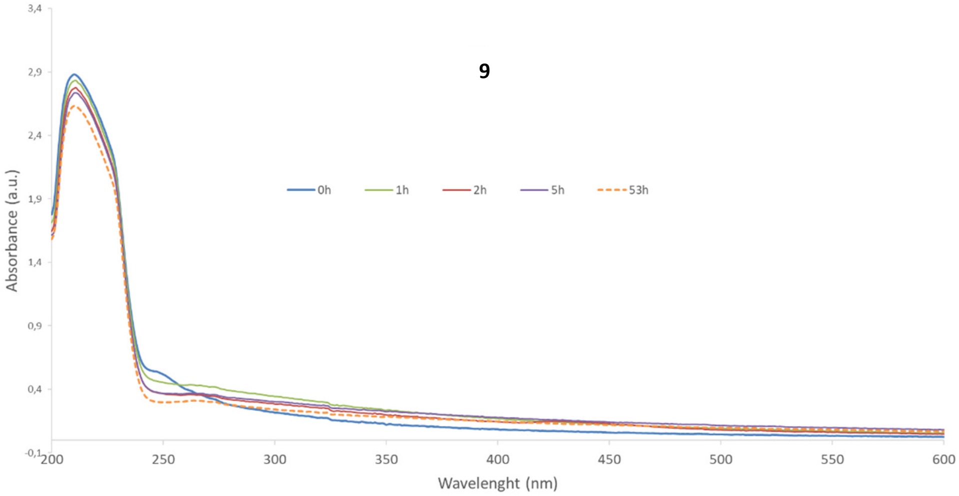

Absorbance (a.u.)
Wavelenght (nm)
0h
1h
2h
5h
53h
-0,1
0,4
0,9
1,4
1,9
2,4
2,9
3,4
200
250
300
350
400
450
500
550
600

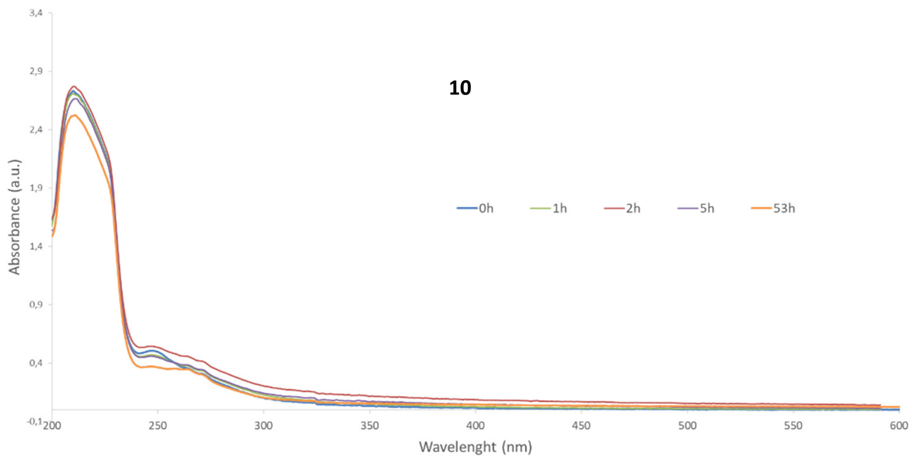

Figure S27. UV-Vis spectra of **1-10** complexes recorded at different times for 24 h to test their stability under physiological conditions. [complex] = 50 μM diluted in PBS at 37 ºC.

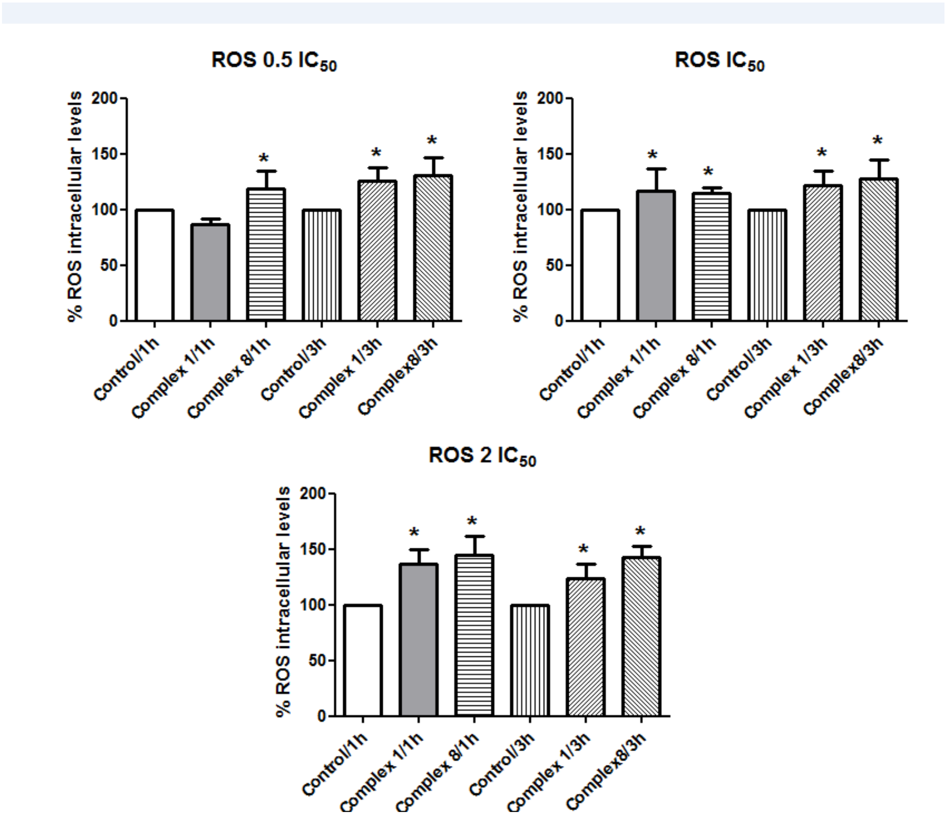

Figure S28. Measurement of intracellular levels of ROS after treatment of Caco-2 cells with complexes **1** and **8** at different concentrations and times. *$p < 0.05$ compared to respective control.
